# A new Miocene skate from the Central Paratethys (Upper Austria): the first unambiguous skeletal record for the Rajiformes (Chondrichthyes: Batomorphii)

**DOI:** 10.1080/14772019.2018.1486336

**Published:** 2018-10-30

**Authors:** Giuseppe Marramà, Ortwin Schultz, Jürgen Kriwet

**Affiliations:** a University of Vienna, Department of Palaeontology, Althanstrasse 14, 1090, Vienna, Austria;; b Natural History Museum Vienna, Department of Geology and Paleontology, Burgring 7, 1010Vienna, Austria

**Keywords:** *Ostarriraja parva* gen. et sp. nov., Rajiformes, Burdigalian, Central Paratethys, Austria, phylogeny

## Abstract

A new fossil skate, *Ostarriraja parva* gen. et sp. nov., represented by a single partial articulated skeleton collected from the early Miocene fish-bearing strata of Upper Austria, is described here in detail. This taxon exhibits a unique combination of skeletal and dental features (e.g. nasal capsules broad and oval; presence of pectoral arch; compound radial articulated with single radial segments in serial fashion; separated pelvic girdle condyles; reduced catenated calcification of radials; about 86 pectoral radials; 20–21 pelvic-fin radials; 65–70 predorsal vertebrae) that clearly support its assignment to a new genus of the order Rajiformes, and the phylogenetic analyses reveal its basal position within the group. The comparison between *Ostarriraja* and the holomorphic batoids from Late Cretaceous of Lebanon traditionally aligned with skates concurs to suggest that this Neogene occurrence represents unquestionably the first known skeletal record for the group. The morphological and phylogenetic affinities of *Ostarriraja* with the living skates suggest a close association of this taxon with the temperate-cold water environments hypothesized for the Central Paratethys during the early Miocene.

http://zoobank.org/urn:lsid:zoobank.org:pub:8BB8F0F3-35C5-47FA-AE3C-2CBF445C4BCA

## Introduction

Skates of the order Rajiformes *sensu* Naylor *et al.* (2012a) are a diverse and well-defined monophyletic group within Batoidea, which includes almost half of all batoid fishes comprising *c.* 290 valid living species arranged in 38 genera. They occur worldwide mainly on continental and insular shelves, from coastal to abyssal depths, and from temperate to cold waters (McEachran & Miyake 1990a, b; Last & Compagno 1999; McEachran & Carvalho 2002; Last *et al.*^2016^). Skates are benthic batoids characterized by a series of derived morphological traits, including oviparous development, alar and malar thorns in adult males, weak spindle-shaped electric organs running bilaterally within the lateral caudal musculature, pelvic fins expanded laterally and usually divided in two lobes, branchial copula with forked anterior expansions, pectoral arch formed by the fusion of suprascapulae to the median crest of the synarcual, and additional unique features of the clasper morphology (e.g. Compagno 1977; McEachran & Miyake 1990a; Herman *et al.* 1996; McEachran & Dunn 1998; Koester 2003; Aschliman *et al.*2012a). The peculiar pelvic girdle morphology of skates is unique among batoids and enables them to perform a form of benthic locomotion called ‘punting’ (e.g. Koester & Spirito 2003; Macesic & Kajiura 2010).

Molecular studies agree with the hypothesis that skates form the sister group of all other batoids (e.g. Aschliman *et al.*2012b; Last *et al.*^2016^), or alternatively are sister to all batoids excluding thornbacks and electric rays (Bertozzi *et al*. 2016), or are even recovered in a large polytomy with the other batoid groups (e.g. Aschliman *et al*. 2012a; Naylor *et al*. 2012a). The familial structure of the skates has been subject to several changes and discussion in recent years (Last *et al*. 2016). The most recent analyses based on mitochondrial and nuclear genes (Naylor *et al*. 2012a, b; Last *et al*. 2016) strongly support the hypothesis that Rajiformes consists of four family-level groups: Anacanthobatidae, Arhynchobatidae, Gurgesiellidae and Rajidae. On the contrary, according to the last morphology-based analysis (McEachran & Dunn 1998) the skates are grouped in a single family (Rajidae) comprising two subfamilies and five tribes: Amblyrajini, Gurgesiellini and Rajini within the subfamily Rajinae, and Arhynchobatini and Riorajini within the subfamily Arhynchobatinae. A monophyletic group including *Fenestraraja*, *Gurgesiella* and *Cruriraja* (Gurgesiellidae) is not recognized in the morphology-based phylogeny of McEachran & Dunn (1998), and the representatives of the family Anacanthobatidae are nested within the subfamily Rajinae.

Although ghost lineages predict that skates should have been already present in the Jurassic (see Bertozzi *et al*. 2016), unambiguous fossil occurrences are only known from the Late Cretaceous (see Siverson & Cappetta 2001; Cappetta 2012). However, very little is known about the evolutionary history of this group, because almost all fossil taxa are represented by isolated teeth, which are phylogenetically poorly informative if not considered in a comprehensive morphological context which includes both dental and skeletal characters (see e.g. Claeson *et al*. 2010; Marramà *et al*. 2018d). Some complete and articulated skeletal remains from the Late Cretaceous deposits of Lebanon have been traditionally assigned to skates, but their placement within the group requires further investigation since they lack most of the diagnostic features of the skates (Siverson & Cappetta 2001; Cappetta 2012; see also Discussion). Although in the Cenozoic articulated batoids were recovered from Palaeogene marine sediments of the Bolca Lagerstätte and Grube Unterfeld, from the freshwater deposits of the Green River Formation, and from the Neogene of SE Asia (Carvalho *et al*. 2004; Hovestadt *et al*. 2010; Marramà *et al*. 2018a, c, d), no articulated fossil skate has been recognized so far. In this paper, we report the first unambiguous holomorphic skate in the fossil record represented by a single specimen from the early Miocene of Upper Austria. The character combination distinguishes the specimen readily from all other skates, allowing its assignment to a new genus within Rajiformes.

## Geological setting

**Figure 1:**
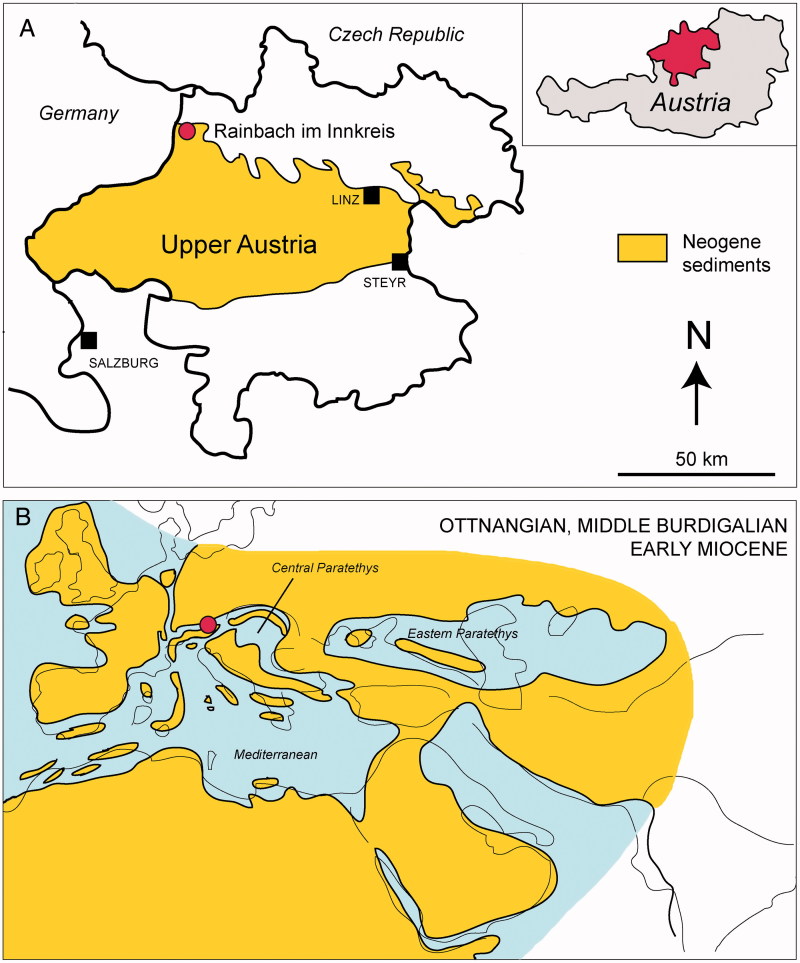
**A,** location and simplified geological map of Upper Austria, based on Rupp *et al.* (2008), showing the position of the locality (red dot) where *Ostarriraja parva* gen. et sp. nov. was found. **B,** palaeogeographical sketch-maps of the Paratethys seas during the Ottnangian, middle Burdigalian, early Miocene showing the possible location of the locality in A, based on Rögl (1998).

The examined specimen comes from the Neogene strata of the North Alpine Foreland Basin (Molasse Basin) in a pit west of the village of Rainbach im Innkreis, about 70 km from Linz, in Upper Austria (Fig. 1A). According to Schultz in Rögl *et al*. (1973) and Brzobohatý & Schultz (1973) the strata of this locality – chosen as faciostratotype – pertain to the Central Paratethys stage Ottnangian, middle Burdigalian, early Miocene (*c.* 18 Ma; see Grunert *et al*. 2010a), whose stratotype has been defined in a pit clay near the village of Ottnang, in Upper Austria (Rögl *et al.*^1973^; Piller *et al.*^2007^; Grunert *et al.*2010a) and corresponding to the planktonic foraminifera Biozone M3 of Berggren *et al.* (1995). During the Ottnangian, the seaway between the Mediterranean and Indian Ocean closed, and the Eastern Paratethys Basin was closed off from the Central Paratethys and Mediterranean, whereas the North Alpine Foreland Basin was part of a marine gateway, known as the Burdigalian Seaway, connecting the western Protomediterranean Sea and the Central Paratethys (Fig. 1B; see Allen *et al*. 1985; Rögl 1998). Micropalaeontological and geochemical proxies indicate that the entire area probably evolved in a context of regional upwelling conditions with strong mixing of surface waters with rising nutrient-rich waters under temperate to cool sea surface temperatures (Bachmann 1973; Grunert *et al*. 2010b), although cold surface temperature cannot be generalized for the entire Central Paratethys. The Ottnangian layers in the area of Rainbach im Innkreis have yielded numerous fish remains, including otoliths, isolated teeth and vertebrae of bony and cartilaginous fishes, indicating a rather rich and diverse fish community, considered by Brzobohatý & Schultz (1973) to be highly endemic. However, the fish fauna forms different associations in different areas of the Central Paratethys, documenting different facies conditions (Brzobohatý & Schultz 1973).

## Material and methods

The study is based on a single specimen in part and counterpart housed in the collections of the Natural History Museum of Vienna (NHMW) and labelled with the repository serial NHMW 2005z0283/0097. The specimen was examined using a stereomicroscope equipped with camera lucida drawing arm. Ultraviolet (UV) light was used to enhance the visibility of some of the skeletal structures, which are difficult to examine under normal light. Teeth of NHMW 2005z0283/0097 were examined and photographed with a Jeol 6400 scanning electron microscope (SEM) at the University of Vienna. Osteological and tooth terminologies mostly follow McEachran & Miyake (1990a), Herman *et al.* (1994, 1995, 1996) and McEachran & Dunn (1998). The term ‘holomorphic’ refers here to being more or less completely preserved and articulated. Following Naylor *et al*. (2012a) and Last *et al*. (2016), the order name Rajiformes is used herein only for skates, including the new taxon described here and the representatives of the families Anacanthobatidae, Arhynchobatidae, Gurgesiellidae and Rajidae, since other batoid lineages traditionally included in Rajiformes (e.g. guitarfishes and sawfishes) are now referred to the separate order Rhinopristiformes.

The phylogenetic analysis is based on the morphological data set of McEachran & Dunn (1998), which in turn is based on the matrix of McEachran & Miyake (1990a). The matrix (see Supplemental material, Appendices A and B) was extended with dental characters of Herman *et al*. (1994, 1995, 1996) and skeletal features provided by Aschliman *et al.* (2012a) and Jeong & Nakabo (2009), which are useful to better define the synapomorphies that distinguish skates from other batoids. The original detailed data matrix of McEachran & Dunn (1998, appendix 3) has been checked and the taxonomic status of the species updated according to the recent classification of Last *et al.* (2016). For example, the species *Anacanthobatis americanus* and *A. foliorostris* of McEachran & Dunn (1998) are included in the genera *Schroederobatis* and *Springeria*, respectively, in our phylogeny. The *Raja* species of the North Pacific and Amphi-American assemblages of McEachran & Dunn (1998) were included in the genera *Beringraja* and ‘*Rostroraja*’, respectively. *Rhinoraja longi* and *R. taranetzi* are synonyms of *Bathyraja taranetzi* and therefore their characters included in the genus *Bathyraja* accordingly. *Notoraja asperula* and *N. spinifera* are species of *Brochiraja*, and *Okamejei australis*, *O. cerva* and *O. lemprieri* are species of *Dentiraja*. *Dipturus* (*Zearaja*) *nasutus* and *Okamejei* (*Orbiraja*) *powelli* were excluded from the analysis because of the lack of several morphological and dental characters useful to define their relationships within Rajiformes. Thus, the data matrix is composed of 74 characters coded for 36 genera, including *Hongeo* from Jeong & Nakabo (2009) and the new fossil taxon described herein. The character matrix was compiled in Mesquite 3.03 (Maddison & Maddison 2008). The phylogenetic analysis was performed with TNT (Tree analysis using New Technology) v. 1.5 (Goloboff *et al.*^2008^). Following McEachran & Dunn (1998) we used the branch-and-bound method, using the swapping algorithm tree bisection reconnection (TBR) via 100 replications of a random stepwise addition, and collapsing trees after the search. All characters are considered unordered and given equal weight, except character (ch.) 13 in order to avoid loss of grouping information. Tree length, Bremer support, and consistency (CI) and retention (RI) indices were subsequently calculated for the 50% majority rule consensus tree retrieved by the analysis. Additional phylogenetic analyses using the same data matrix but different algorithms, or excluding dental characters, were excuted in TNT for cross-checking results obtained from the main analysis.

## Comparative material

Extant taxa: *Amblyraja* sp., UNIVIE EMRG-Chond-H1: the specimen was cleared and stained at the Department of Palaeontology, University of Vienna following the procedure used by Walker & Kimmel (2007); *Bathyraja kincaidii*, VNHM-ID 8081 and VNHM-ID 7514: CT-scan created by #ScanAllFishes, copyright CC-BY-NC, Virtual Natural History Museum (www.vnhm.eu); *Raja clavata*, UNIVIE EMRG-H-2: the tail of the specimen was investigated non-invasively with a (micro-computed tomography (micro-CT) device SkyScan/Bruker 1173 at the Department of Palaeontology, University of Vienna. The scan settings were 50 kV and 160 μA, with a scan resolution of 7.86 μm. The processing of the .tiff image stacks was performed with Amira v. 5.4.1 (Visualization Sciences Group); *Raja rhina*, VNHM-ID 8081: CT-scan created by #ScanAllFishes, copyright CC-BY-NC, Virtual Natural History Museum (www.vnhm.eu).

Fossil taxa: *Cyclobatis oligodactylus*: two specimens, MNHN F.HDJ504 and MNHN F.HDJ505; *Cyclobatis major*: one specimen, MNHN HAK555; *Rajorhina expansa*: one specimen, MNHN HAK561 and MNHN HAK558 (part and counterpart).

## Institutional abbreviations

**MNHN**: Museum national d’Histoire naturelle, Paris, France; **NHMW**: Naturhistorisches Museum Wien, Austria; **UNIVIE EMRG**: Ichthyological collection of the Department of Palaeontology, University of Vienna, Austria; **VNHM**: Virtual Natural History Museum (www.vnhm.eu).

## Systematic palaeontology

Class **Chondrichthyes** Huxley, 1880Superorder **Batomorphii** Cappetta, 1980Order **Rajiformes** Berg, 1937*sensu* Naylor *et al.* (2012a)Family ***incertae sedis***Genus ***Ostarriraja*** gen. nov.

**Derivation of name.** The genus name is derived from the Old High German word ‘Ostarrîchi’ for Austria, meaning ‘eastern realm’, in allusion to its provenance, and the Latin word ‘raia’ or ‘raja’ in Medieval Latin (gender feminine), meaning ‘ray’, referring to its systematic relationships.

**Type species.***Ostarriraja parva* sp. nov.

**Diagnosis.** A skate characterized by the following combination of morphological and dental characters: nasal capsules broad and oval, possibly without basal fenestrae; distance between pro- and mesocondyles less than distance between meso- and metacondyles; about 86 pectoral radials of which 33 are propterygial, 10 mesopterygial, 32 metapterygial and 11 directly articulated to scapulocoracoid between meso- and metacondyles; about 20–21 pelvic-fin radials; lateral prepelvic processes of pelvic girdle moderately long; pelvic fin lobes continuous, without a gap in the arrangement of radials between the compound radial and basipterygial radials; 65–70 predorsal vertebrae; tooth crown massive, semi-oval in occlusal view, with an arched labial edge and a lingual one with a small medial protuberation; well-marked transverse cutting crest; mesial and distal cutting edges concavely arched and reaching the margins of the crown; labial cutting edge absent; apron, uvula and crown ornamentation absent; holaulacorhizid teeth with unequally developed massive root lobes; root as wide as the crown; root stem relatively high; pair of margino-lingual foramina on root; root coating well marked; cross-type tail thorns, displaced in three antero-posteriorly directed parallel rows.

**Remarks.** In a preliminary report, Brzobohatý & Schultz (1973, p. 657) identified tentatively this nearly complete and articulated batoid specimen from the early Miocene of Rainbach im Innkreis, Upper Austria, as an indeterminate species of the stingray genus *Dasyatis* of the batoid order Myliobatiformes, based on the overall shape of the disc and the presence of a long whip-like tail. Our in-depth anatomical investigation of the type material originally described by Brzobohatý & Schultz (1973) excludes the hypothesis that *Ostarriraja* pertains to Myliobatiformes because of the absence of important diagnostic characters of the stingrays, including e.g. a second (thoracolumbar) synarcual, serrated tail stings, distance between pro- and mesocondyle larger than meso- and metacondyle, etc. (see Carvalho *et al.*^2004^; Aschliman *et al.*2012a; Marramà *et al.*2018a). On the contrary, the recognition of several diagnostic skeletal and dental characters of the skates supports the assignment of *Ostarriraja* to the order Rajiformes (see Description and Discussion).

**Figure 2:**
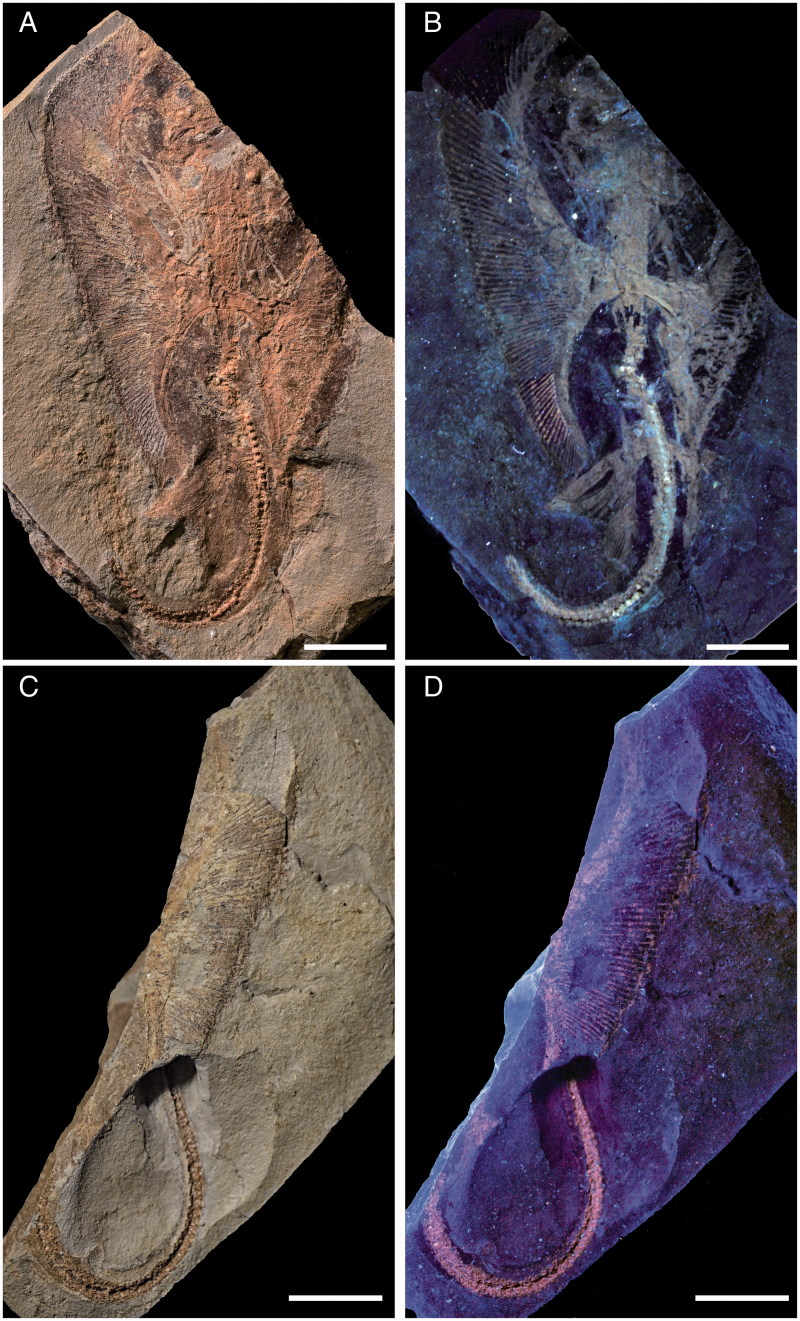
*Ostarriraja parva* gen. et sp. nov. from the early Miocene of Upper Austria. **A,** NHMW 2005z0283/0097a, holotype under normal light; **B,** NHMW 2005z0283/0097a, holotype under UV light; **C,** NHMW 2005z0283/0097b, holotype, counterpart under normal light; **D,** NHMW 2005z0283/0097b, holotype, counterpart under UV light. Scale bars: 10 mm.

**Figure 3:**
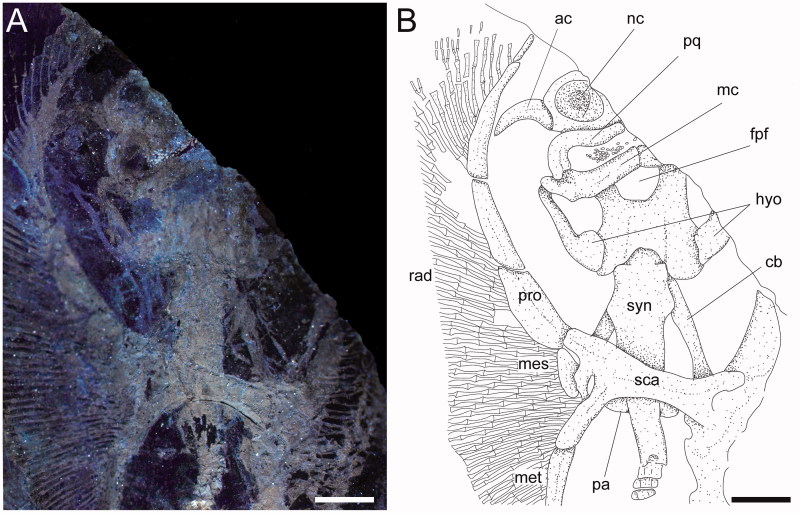
*Ostarriraja parva* gen. et sp. nov. from the early Miocene of Upper Austria, holotype, NHMW 2005z0283/0097. **A,** close-up of the head and pectoral region of the holotype under UV light; **B,** restoration. Abbreviations: ac, antorbital cartilage; cb, 5th ceratobranchial; fpf, fronto-parietal fontanelle; hyo, hyomandibula; mc, Meckel’s cartilage; mes, mesopterygium; met, metapterygium; nc, nasal capsule; pa, pectoral arch; pq, palatoquadrate; pro, propterygium; rad, radials; syn, synarcual. Scale bars: 5 mm.

***Ostarriraja parva*** sp. nov.(Figs 2, 3, 5–7)

1973 *Dasyatis* (?) sp.; Schultz in Brzobohatý & Schultz: 657, pl. 1, fig. 10.

1973 *Dasyatis* (?) sp.; Schultz in Rögl, Schultz, & Hölz: 154.

2013 *Dasyatis* sp.; Schultz: 103, pl. 30, fig. 1.

**Derivation of name.** From the Latin word *parvus* meaning ‘small’, referring to the small size of the specimen; gender feminine.

**Holotype.** NHMW 2005z0283/0097, nearly complete articulated skeleton in part and counterpart preserved in a slab having length and width of 98.3 and 64.1 mm, respectively (Fig. 2).

**Type locality and horizon.** Rainbach im Innkreis, Upper Austria, Austria; Ottnangian, middle Burdigalian, early Miocene (*c.* 18 Ma; see Grunert *et al*. 2010a). 

**Diagnosis.** As for the genus.

**Description.** Measurements and body proportions are difficult to establish since the specimen does not preserve the anterior-most portion of the body (including the rostral cartilage), the posterior-most tip of the tail, or the distal-most segments of the pectoral radials, rendering it difficult to detect the exact outline, the width of the pectoral disc and the total length of the specimen. However, the skate is quite small (possibly reaching a length of 12–14 cm in life) and is preserved as part and counterpart in a subrectangular slab having a length and width of 98.3 and 64.1 mm, respectively (Fig. 2). The small size of the specimen, the morphology of teeth, the poor mineralization of some skeletal structures, and the absence of claspers suggest that the specimen might represent a juvenile female skate, although the almost complete mineralization of the synarcual might indicate a late juvenile or even subadult. The specimen is displayed in ventral view, as suggested by the exposure of jaws and teeth, and the scapulocoracoid bar of the pectoral girdle lying ventrally to the vertebral axis. The body is dorso-ventrally compressed, and the disc appears largely free of dermal denticles and thorns, at least ventrally. However, multiple rows (at least three) of thorns are present along the tail, which is long and robust, but not slender and whip-like as in skates of the family Anacanthobatidae (see Hulley 1973; Last *et al.*^2016^). Several parts of the skeleton show the typical prismatic calcification of elasmobranch fishes (Dean & Summers 2006) and are highly visible in UV light (Figs 2, 3, 6). However, some of the skeletal elements, such as the distal-most segments of the pectoral radials, are not preserved, suggesting that they were still poorly or not at all mineralized. The number of dorsal fins, typically present near the extremity of the tail in skates (e.g. Last *et al.*^2016^) is unknown in *Ostarriraja*, because the tip of the tail is not preserved.

**Figure 4:**
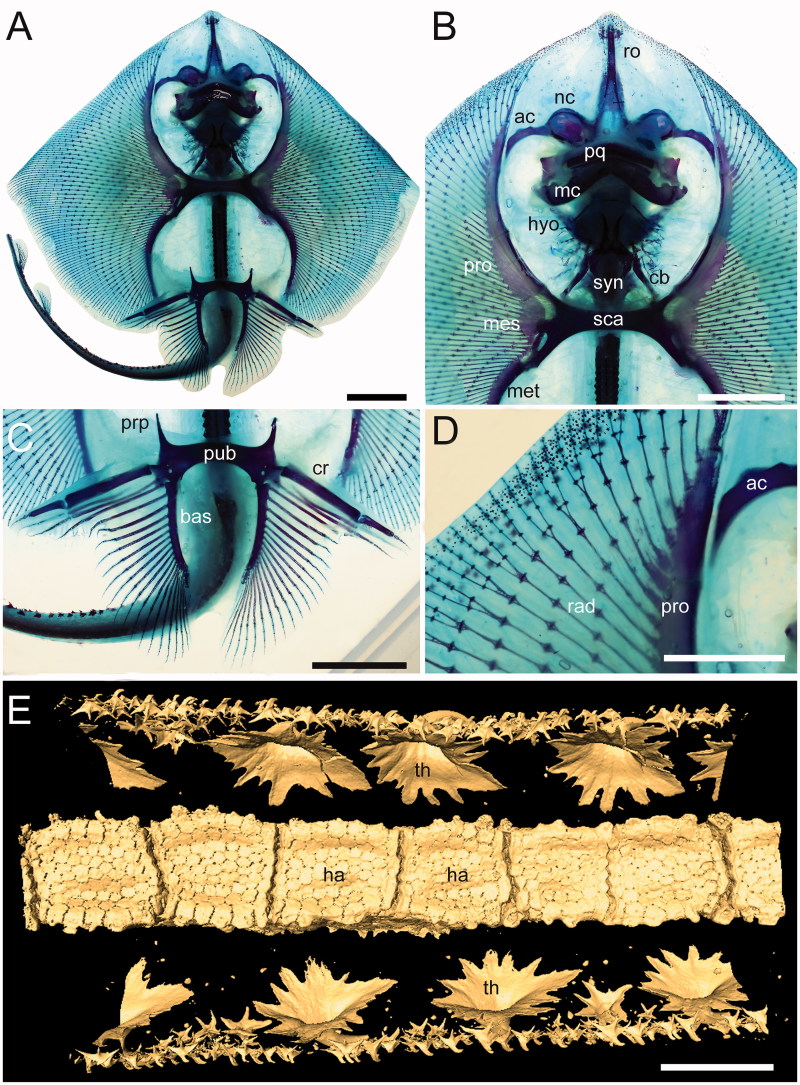
Anatomical details of some of the living skate representatives used for comparisons. **A**–**D,***Amblyraja* sp., UNIVIE EMRG-Chond-H1, cleared and stained specimen; **E,***Raja clavata* Linnaeus, 1758, UNIVIE EMRG-H2, micro-CT scan of the tail; **A,** overall view of the specimen in ventral view; **B,** close-up of the head and pectoral region; **C,** close-up of the pelvic girdle and fins; **D,** detail of the propterygial radials near the head region; note their reduced catenated calcification; **E,** detail of the vertebral column on the tail of UNIVIE EMRG-H2 in ventral view, showing the prismatic calcification of the cartilage forming the haemal arches. Abbreviations: ac, antorbital cartilage; bas, basipterygium; cb, 5th ceratobranchial; cr, compound radial; ha, haemal arches; hyo, hyomandibula; mc, Meckel’s cartilage; mes, mesopterygium; met, metapterygium; nc, nasal capsule; pq, palatoquadrate; pro, propterygium; prp, prepelvic process; pub, puboischiadic bar; ro, rostral cartilage; sca, scapulocoracoid; syn, synarcual; th, thorns. Scale bars: A–C = 20 mm; D = 10 mm; E = 5 mm.

**Cranium.** Although the cranium is only partially preserved, it appears antero-posteriorly elongated, longer than wide, with its greatest width at the level of the nasal capsules (Fig. 3). The rostral cartilage is not preserved. Only one of the two nasal capsules is exposed. It appears broad and oval without the kidney-shaped basal fenestra on its antero-medial aspect typically present in *Psammobatis*, *Irolita*, *Pseudoraja*, *Pavoraja*, *Notoraja*, *Fenestraja* and *Gurgesiella* (see McEachran & Miyake 1990a; McEachran & Dunn 1998) but mostly resembling the condition seen in *Amblyraja* (Fig. 4A, B). Since the specimen is exposed in ventral view, the presence and morphology of the preorbital processes remain ambguous (= preorbital flanges of McEachran & Miyake 1990a). The neurocranium is narrower at the level of the orbital region. The otic capsules are short and robust. Although the specimen shows the ventral side in the main slab, it is possible to recognize (possibly due to taphonomic compression) the outline of the posterior portion of the fronto-parietal fontanelle, whose posterior margin appears concave and does not display any indentation. The antorbital cartilage is massive, unbranched and arched (Fig. 3), and its maximum width is at the level of the articulation with the postero-lateral aspect of the nasal capsule. It tapers distally and extends laterally articulating with the third segment of the propterygium, resembling the condition of skates (Fig. 4B).

**Jaws and hyoid arch.** Only the right hemi-jaws are preserved. The palatoquadrate is broadly arched, labio-lingually compressed, and slightly smaller and narrower than the Meckel’s cartilage (Fig. 3). The palatoquadrate slightly tapers towards the symphysis, and possesses a strong condyle that articulates with the Meckel’s cartilage at the mandibular articular fossa. The Meckel’s cartilage is stouter and broader than the palatoquadrate. Its antimeres are robust, not tapering, and are separated at the symphysis. There are no labial cartilages. The hyomandibulae are narrow and elongate, enlarged and stout proximally at the articulation with the otic region of the neurocranium, and tapering distally towards their articulation with the Meckel’s cartilage. With the exception of only the fifth pair of ceratobranchials, the ventral gill arch skeleton of *Ostarriraja* is not preserved. However, the fifth ceratobranchials are long and straight, and articulate with the anterior margin of scapulocoracoid.

**Figure 5:**
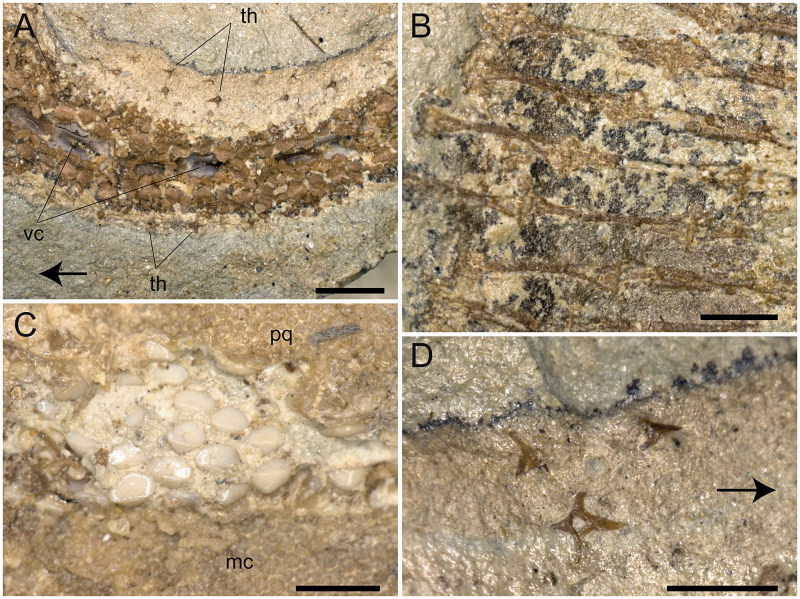
*Ostarriraja parva* gen. et sp. nov. from the early Miocene of Upper Austria, holotype, NHMW 2005z0283/0097. **A,** close-up of the tail with the anterior part on the left side; **B,** detail of the radials in the pectoral disc; **C,** close-up of the teeth; **D,** detail of some tail thorns. Abbreviations: mc, Meckel’s cartilage; pq, palatoquadrate; th, thorns; vc, vertebral centra. The arrows indicate the rostral direction. Scale bars: A–C = 1 mm; D = 0.5 mm.

**Synarcual and vertebral column.** The synarcual cartilage (Fig. 3) is strongly calcified and tube-shaped, and its morphology is consistent with that of skates (see Claeson 2008, 2011). Anteriorly, the synarcual articulates with the occipital condyles of the chondrocranium through a subrectangular synarcual lip which rests inside the foramen magnum of the chondrocranium, a condition considered derived in skates (Aschliman *et al*. 2012a). The two lateral occipital cotyles of the synarcual articulate with the occipital condyles of the chondrocranium. The synarcual extends posteriorly to the level of the shoulder girdle. The pectoral arch, formed by the fusion of suprascapulae to the dorsal median crest of the synarcual in skates (Claeson 2008; Aschliman *et al*. 2012a), is partially visible posterior to the scapulocoracoid bar. However, the dorsal median crest of the synarcual and the lateral stays are not exposed in our specimen. It is not possible to detect the number of fused vertebrae that form the synarcual, or the spinal nerve foramina. Two or three unfused individual vertebral centra can be seen near the posterior margin of the synarcual. At least 13–15 trunk vertebrae (from the first distinguishable centrum to the anterior margin of the puboischiadic bar) can be recognized. About 50 vertebrae are visible from the anterior margin of the puboischiadic bar to the last portion of the tail although this number was originally higher since the distal tip of the tail is not preserved. However, it is likely that the total number of predorsal vertebrae might have been about 65–70. The vertebral centra are strongly calcified, subrectangular in shape and antero-posteriorly elongated. The vertebrae of the tail appear to be surrounded by small calcified tesserae of polygonal shape (Fig. 5A), which probably represent the tesserae that form the prismatic calcification of the modified neural and haemal arches in modern skates (Fig. 4E). Ribs are absent.

**Pectoral fins and girdle.** The scapulocoracoid is formed by a single straight and robust transverse structure, located just ventral to the synarcual arch (Fig. 3). The scapulocoracoid articulates anteriorly with the fifth pair of ceratobranchials. The lateral aspect of the scapulocoracoid appears to be large, and the distance between pro- and mesocondyles is less than the distance between meso- and metacondyles, contrary to some skates like *Amblyraja*, in which the distance between condyles is about equal (Fig. 4B). The presence of the bridge and the postventral fenestra cannot be detected in *Ostarriraja*. The propterygium is long and arched, and gradually tapers distally. It is segmented and at least four segments can be recognized; the proximal one is enlarged and articulates with the procondyle on the anterior portion of the lateral margin of the scapulocoracoid. The third propterygial segment articulates mesially with the antorbital cartilage of the neurocranium. Due to the incomplete preservation of the anterior portion of the body it is not clear whether the propterygium extends anteriorly to the rostral node. The mesopterygium is small, shorter than the pro- and metapterygium. It is a single ovoid element and its external margin is more or less straight to slightly convex, apparently not fused to the radials. The metapterygium is arched and its length is less than that of the propterygium. The metapterygium gradually tapers distally, where it segments at least once. There are about 86 pectoral radials. Of these, 33 are propterygial, 10 mesopterygial and 32 metapterygial. Moreover, about 11 radials directly articulate with the scapulocoracoid between the mesopterygium and metapterygium, resembling the typical condition of skates, *Pristis*, panrays and guitarfishes (Garman 1913, pls 64 and 65; Nishida 1990, fig. 32; McEachran *et al*. 1996, fig. 9; Aschliman *et al.*2012a). Each pectoral radial is composed of at least five segments. However, since the external margin of the disc is incompletely preserved, possibly due to the incomplete mineralization of the distal-most radial cartilages in this presumably late juvenile specimen, it is possible that the number of segments was much higher. The anterior-most propterygial radials bifurcate distally at least once. The radials of *Ostarriraja* are calcified in chain-like patterns (Fig. 5B), forming the so-called ‘catenated calcification’ typical of batoids with undulatory swimming mode, including skates and most of the benthic stingrays (Schaefer & Summers 2005). Moreover, as observed in modern skates (Fig. 4D; see also Schaefer & Summers 2005, fig. 2), the calcification is further reduced in *Ostarriraja*, since it consists of a single chain on the dorsal and ventral sides in all radials.

**Figure 6:**
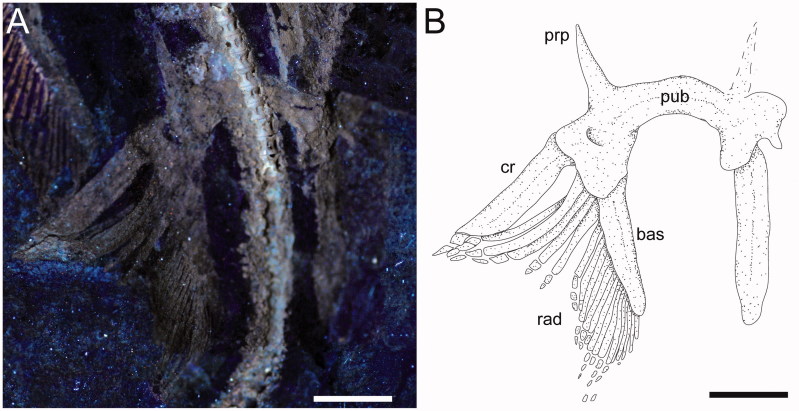
*Ostarriraja parva* gen. et sp. nov. from the early Miocene of Upper Austria. **A,** NHMW 2005z0283/0097, close-up of the pelvic girdle and fins of the holotype under UV light; **B,** restoration. Abbreviations: bas, basipterygium; cr, compound radial; prp, prepelvic process; pub, puboischiadic bar; rad, radials. Scale bars: 10 mm.

**Pelvic fins and girdle.** As observed in modern skates (Fig. 4C; see also Compagno 1999; Holst & Bone 1993, fig. 1; Lucifora & Vassallo 2002, fig. 2), the pelvic fins of *Ostarriraja* are typically bilobed, characterized by the presence of distinct anterior and posterior lobes (Fig. 6). The anterior lobe is supported by a rod-like compound radial (articulated proximally to the radial condyle of pelvic girdle and distally in serial fashion with the proximal radial, which in turn is articulated to the distal radial) and three or four most anterior radials arising directly from the puboischiadic bar. The posterior lobe, conversely, is supported by the long basipterygium, articulating with the basal condyle of the pelvic girdle, which sustains 16 basipterygial radials. Pelvic girdle condyles for the compound radial and the basipterygium are therefore distinctly separated, and the pelvic fins include about 20–21 total radials each. The presence of anterior radials arising directly from the puboischiadic bar clearly distinguish the morphology of the pelvic fins of *Ostarriraja* from those of *Cruriraja*, *Schroederobatis* and *Springeria*, which are unique among skates in the absence of radials in the proximal section of the basipterygium and radials arising directly from the puboischiadic bar, therefore leaving a gap in the distribution of the pelvic-fin radials (Bigelow & Schroeder 1948; McEachran & Miyake 1990a, fig. 12; McEachran & Dunn 1998). The puboischiadic bar is robust and wide, with a slightly convex anterior margin, although we do not exclude that taphonomy might have influenced, at least in part, the preservation of this structure, as well as the low-quality preservation of the right side of the pelvic fins. The presence of the puboischiadic foramina is difficult to detect. The prepelvic processes are moderately long, straight and pointed, extending anteriorly beyond the level of the posterior tip of metapterygia. However, the prepelvic processes are considerably shorter than one-half the width of the puboischiadic bar, distinguishing them from those of *Psammobatis* and *Sympterygia* (longer than one-half width of the puboischiadic bar) or from *Schroederobatis* and *Springeria* (long with forked tips) (McEachran & Miyake 1990a, figs 8 and 12; McEachran & Dunn 1998). Claspers are not present, corroborating the hypothesis that NHMW 2005z0283/0097 represents a young female.

**Dentition.** The teeth of *Ostarriraja* are small (*c*. 400–500 μm in crown width) and arranged in numerous rows (Fig. 5C). The dentition is of crushing type with a gradient monognathic heterodonty. The teeth are uncusped and slightly decrease in size distally. Sexual and ontogenetic heterodonties are unknown because only a single specimen is available. The specimen shows only three or four tooth files on each jaw, resembling the condition of the early tooth development in skates (see Underwood *et al*. 2015).

**Figure 7:**
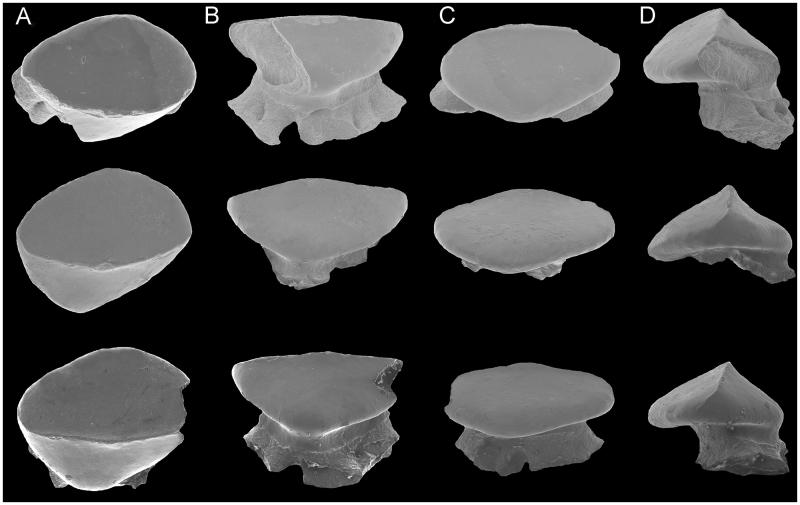
*Ostarriraja parva* gen. et sp. nov. from the early Miocene of Upper Austria. Three isolated teeth from the holotype NHMW 2005z0283/0097 in **A,** occlusal; **B,** lingual; **C,** labial; and **D,** lateral view. Scale bar: 200 μm.

The crown is massive, semi-oval in occlusal view, and longer mesio-distally than labio-lingually, with an arched labial edge and a lingual one with a small medial protuberance (Fig. 7). The teeth show a well-marked transverse cutting crest that separates the labial and lingual crown surfaces. The mesial and distal cutting edges are concavely arched and reach the margins of the crown. A labial cutting edge is absent. There is no strong cusp but the crown bears a poorly developed, semi-centrally situated and erected cone. The apron, uvula and crown ornamentation are absent. The lingual surface is weakly convex. The root is obliquely directed lingually. The root is massive, as wide as the crown but not protruding below the crown in occlusal view, relatively high and more or less oval in cross section. The root is holaulacorhize and bilobate, with unequally developed massive root lobes that widen basally to form a large base with slightly undulated margins. However, we do not exlude that the presence of unequally developed root lobes could be due to the juvenile stage of the specimen or the position of the teeth within the jaws. A pair of margino-lingual foramina is present. Their presence might indicate the presence of fusion of root lobes, although we do not exclude, again, that fusion might be related to the juvenile stage. The collar (= root coating of Herman *et al.* 1996) is very distinct and covers the upper part of the root stem.

**Squamation.** The surface of the disc of *Ostarriraja* appears to be largely free of dermal denticles. Thorns on the dorsal surface of the nuchal and scapular region of the disc, as well as thorns along the dorsal midline of the disc, appear absent, although this might be due to the ventral exposure of the specimen, which prevents identification of the dorsal surface of the disc. Malar and alar thorns also appear to be absent. However, it must be emphasized that malar and alar thorns are only present in mature male skates (McEachran & Konstantinou 1996; McEachran & Dunn 1998), and their presence in *Ostarriraja* is very unlikely due to the sex (female) and late juvenile stage of the fossil specimen (development of denticles proceeds caudo-rostrally in skates; Miyake *et al.*^1999^). However, multiple rows of thorns are present along the entire dorsal margin of the tail (Fig. 5A). Thorns on the tail occur in three originally equally spaced parallel rows (one mediocaudal and two laterocaudal) formed by at least 35 similar-sized and similar-shaped pungent thorns each. In dorsal view (Fig. 5D) thorns are bilateral symmetric with four strongly developed elongated ridges forming right angles to each other, typical of the cross-type morphology of dermal denticles of some skates (Gravendeel *et al*. 2002). The anterior elongate ridge is larger than the posterior one. The crown of the thorn is hook-like and obliquely implanted on the basal plate. The cusp of the crown clearly overshoots the basal plate posteriorly and the crown is almost as long as the basal plate, resembling the condition of thorns in *Rajella lintea* (= *Dipturus linteus* of Gravendeel *et al*. 2002).

## Phylogenetic analysis

**Figure 8:**
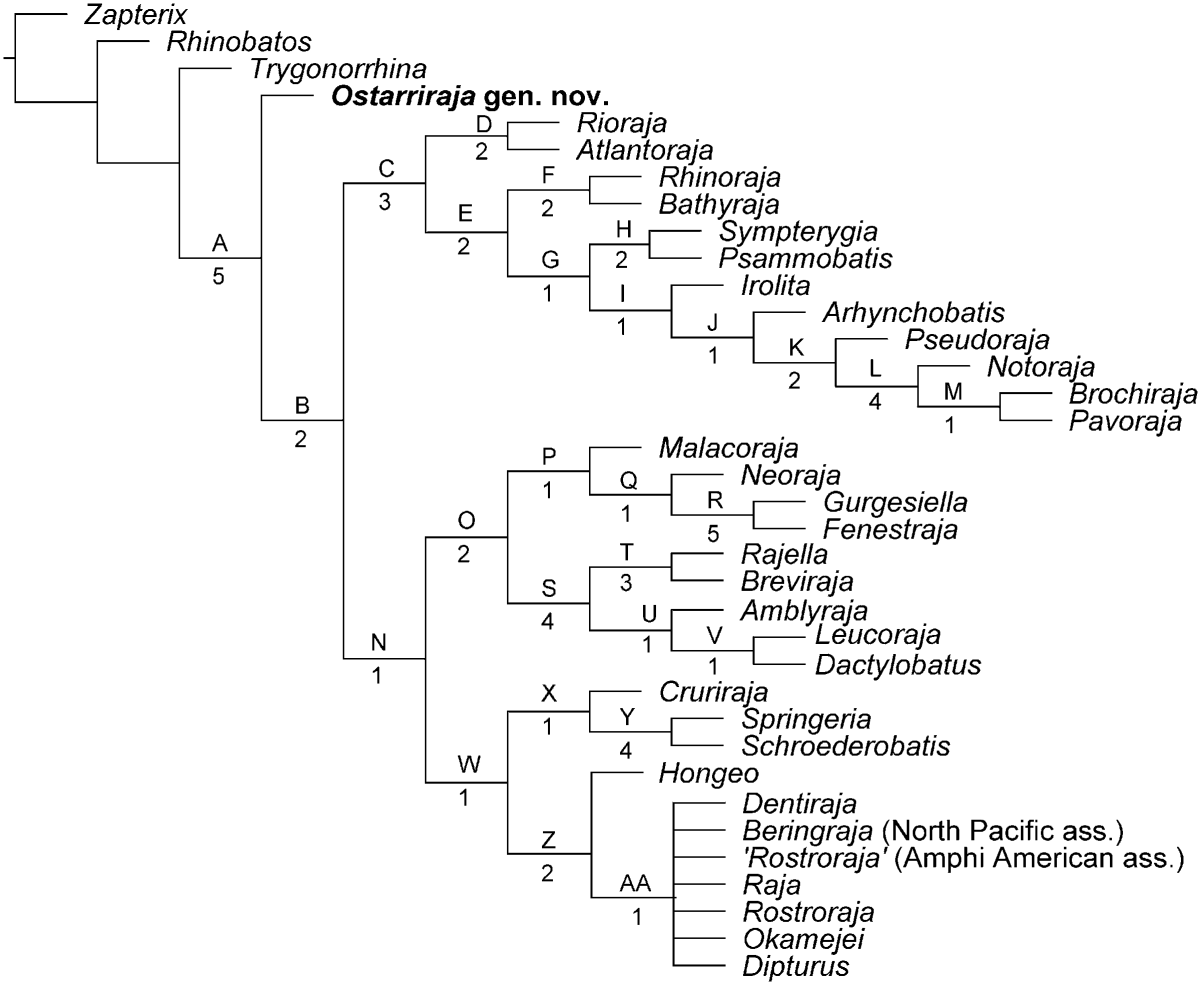
The 50% majority rule consensus tree detected from the analysis of 74 morphological characters coded for 36 taxa showing the hypothetical relationships of *Ostarriraja parva* gen. et sp. nov. within Rajiformes. The list of synapomorphies on each node (capital letters) is given in Supplemental material, Table S1. Numbers below the nodes indicate the Bremer support.

The analysis of 74 morphological characters coded for 36 taxa produced 90 most parsimonious trees that were used to build the 50% majority rule consensus tree having a length of 212 steps, a CI of 0.46 and an RI of 0.76 (Fig. 8). Elevated homoplasy and the relatively high RI suggest that there is a phylogenetical signal in the characters, supporting the hypothesis that considerable parallelisms occur in the morphological evolution within the group (McEachran & Dunn 1998). A complete list of synapomorphies for each node is given as Supplemental material in Table S1. The recovered tree is consistent, at least in part, with the morphology-based phylogeny of McEachran & Dunn (1998), but not with the most recent molecular analyses (e.g. Naylor *et al.*2012a, b; Last *et al.*^2016^). Considering the presence of the pectoral arch, a compound radial that is rod-like and articulated with single radial segments in serial fashion, and separated pelvic girdle condyles to be diagnostic for skates (Garman 1913; Holst & Bone 1993; Lucifora & Vassallo 2002; Claeson 2008, 2011; Aschliman *et al*. 2012a), *Ostarriraja* gen. nov. is recovered herin as the basal-most rajiform, and the monophyly of the whole group is strongly supported (Bremer value 5), by five synapomorphies: nasal capsules broad and oval (ch. 29[1]); uvula absent (ch. 65[1]); suprascapulae fused to the median crest of the synarcual, forming the pectoral arch (ch. 71[1]); rod-like compound radial articulated with single radial segments in serial fashion (ch. 72[1]); separated pelvic girdle condyles (ch. 73[1]). In McEachran & Dunn (1998) the monophyly of Rajiformes was corroborated by the presence of at least six different characters (i.e. oviparous development; alar and/or malar thorns present in mature males; electric organs in lateral tail musculature; second hypobranchial cartilage fused with basibranchial copula; anterior portion of second hypobranchial cartilage absent and proximal section of second hypobranchial cartilage not articulating with second ceratobranchial cartilage; clasper skeleton with dorsal terminal cartilage on dorsal aspect of clasper). However, these characters, although included in the matrix, are not supportive of Rajiformes in our phylogeny, since their presence in the single specimen of *Ostarriraja* is impossible to establish (although highly probable). The phylogeny of Aschliman *et al.* (2012a) recovered the presence of osteodentine to be supportive of the monophyly of skates. However, true osteodentine in large teeth was only detected in the roots of *Rhinoraja* and *Rostroraja* (Herman *et al*. 1994, 1995, 1996) and its presence is therefore not supportive of the clade in our phylogeny. Although a synarcual lip resting inside the foramen magnum was detected as supportive of the skates by Aschliman *et al.* (2012a) and is also present in *Ostarriraja*, it was not included in our phylogenetic analysis since this character appears to be also present in *Pristis* (Rhinopristiformes; see Aschliman *et al*. 2012a), and therefore the inclusion of a homoplastic character in our phylogeny might have supported erroneously the monophyly of skates.

Our phylogenetic analysis recovered a dichotomous nature of living skates which is consistent, at least in part, with the morphological hypothesis of McEachran & Miyake (1990a) and McEachran & Dunn (1998). However, some polytomies detected in the previous analyses are now resolved, possibly because of the inclusion of new dental characters. The monophyletic status of living skates seems to be achieved by the acquisition of the secondary hemiaulacorhizy (ch. 63[1]), loss of the root coating (ch. 69[0]), and fusion of radials to mesopterygium (ch. 74[2]). The living skates are grouped in two main monophyletic groups that correspond to the subfamilies Arhynchobatinae and Rajinae as recognized by McEachran & Dunn (1998). The monophyly of Arhynchobatidae *sensu* Naylor *et al.* (2012a) was therefore detected in our study, but not that of Rajidae *sensu* Naylor *et al.* (2012a). Although the monophyly of Anacanthobatidae *sensu* Last *et al*. (2016) (including here *Springeria* and *Schroederobatis*) has been detected in our analysis, this clade is nested within Rajidae. Moreover, Gurgesiellidae of Last *et al.* (2016) (including *Cruriraja*, *Gurgesiella* and *Fenestraja*) has not been recognized as a monophyletic group herein.

The monophyly of Arhynchobatidae (including *Rioraja*, *Atlantoraja*, *Rhinoraja*, *Bathyraja*, *Sympterygia*, *Psammobatis*, *Irolita*, *Arhynchobatis*, *Pseudoraja*, *Notoraja*, *Brochiraja* and *Pavoraja*) is well supported herein (Bremer value 3) by three synapomorphies: basihyal with lateral projections (ch. 33[1]); clasper glans with component projection (ch. 45[1]); principal tooth cusp oblique (ch. 59[1]). All remaining skates form a monophyletic group (Rajidae herein) supported by five synapomorphies: presence of malar thorns (ch. 23[1]); scapulocoracoid without anterior bridge (ch. 34[2]); clasper glans distally expanded (ch. 40[1]) and with component rhipidion (ch. 41[1]); electrocytes with cortical processes (ch. 57[1]). It is worthy to note that the presence of malar thorns, traditionally considered diagnostic for all skates, has been recognized as supportive only for the family Rajidae. This family consists of three clades (Rajinae + (Amblyrajinae + Gurgesiellinae)) whose relationships have been partially resolved with respect to the phylogeny of McEachran & Dunn (1998). The clade Rajinae (Rajini of McEachran & Dunn 1998) is herein supported by three synapomorphies: dorsal surface largely naked (ch. 11[1]; crown of alar thorns with barb (ch. 20[0]); narrow and rectilinear nasal capsules (ch. 29[0]). *Springeria* and *Schroederobatis* (family Anacanthobatidae of Last *et al.*^2016^) are sister to *Cruriraja*, and these three genera together are sister to a clade including *Hongeo* which is sister to a large polytomy including *Dipturus*, *Dentiraja*, *Beringraja*, *Rostroraja*, *Okamejei* and *Raja*.

The monophyly of the family Gurgesiellidae *sensu* Last *et al.* (2016), including *Gurgesiella*, *Fenestraja* and *Cruriraja*, is not resolved in our phylogeny, with these taxa being polyphyletic. On the contrary, *Gurgesiella* and *Fenestraja*, along with *Neoraja* and *Malacoraja*, form here a monophyletic group (Gurgesiellini of McEachran & Dunn 1998), having a narrow internasal plate (ch. 31[1]). The remaining rajids (*Rajiella*, *Breviraja*, *Amblyraja*, *Leucoraja* and *Dactylobatus*) form a monophyletic group (Amblyrajinae herein) that is well supported (Bremer value 4) by six synapomorphies (see Supplemental material, Table S1).

**Figure 9:**
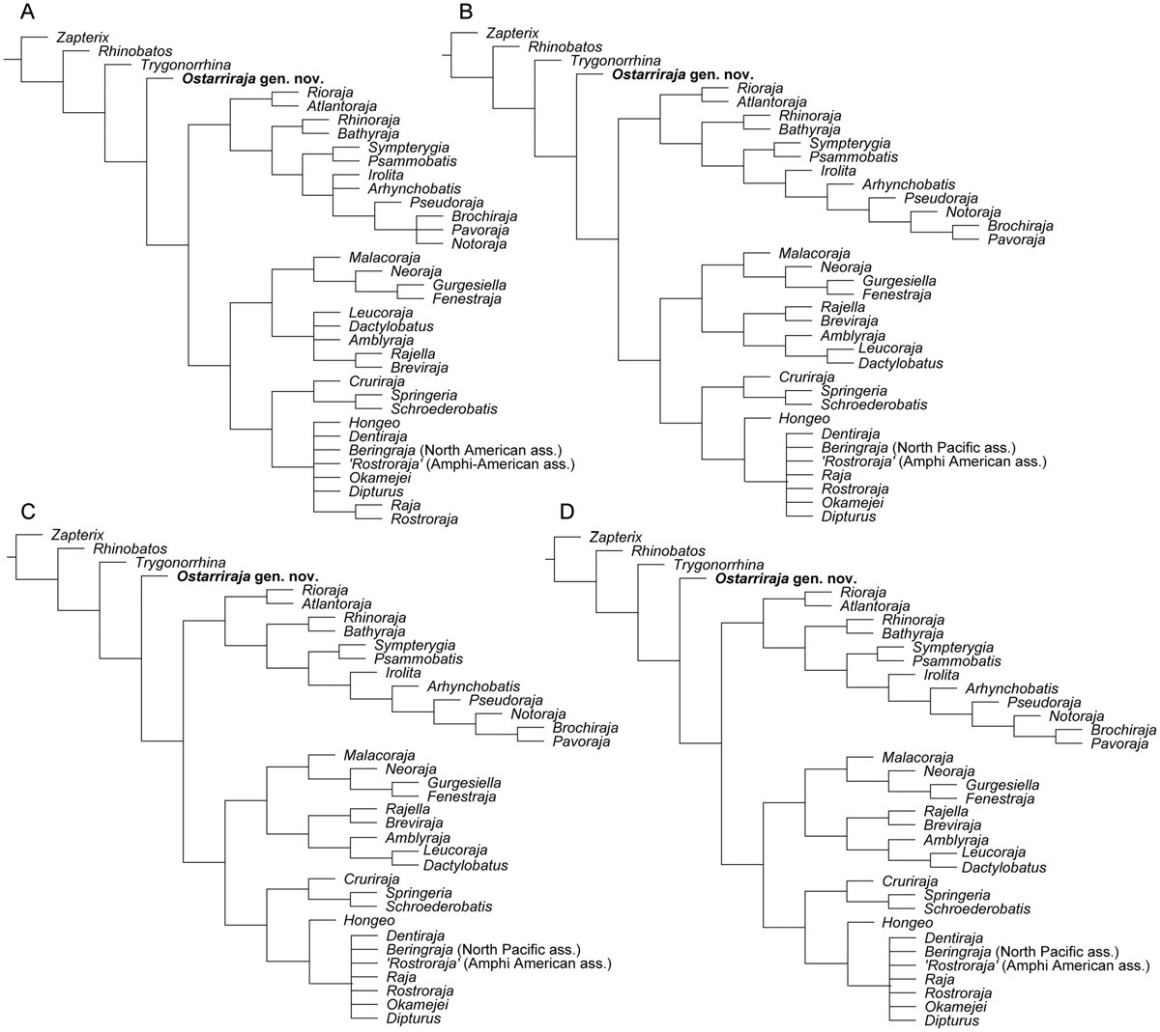
Additional phylogenetic analyses executed in TNT 1.5 for cross-checking results obtained from the main analysis. **A,** 50% majority rule consensus tree (tree length =148 steps, CI =0.57, RI =0.84) detected by excluding dental characters (59 to 70 of Supplemental material, Appendix A) from the main analysis; **B,** 50% majority rule consensus tree (tree length = 212 steps, CI = 0.46, RI = 0.76) detected using the New Technology Search analysis; **C,** 50% majority rule consensus tree (tree length = 212 steps, CI = 0.46, RI =0.76) detected by branch-and-bound method, using the swapping algorithm tree bisection reconnection (TBR) via 1000 replications; **D,** 50% majority rule consensus tree (tree length = 212, CI = 0.46, RI = 0.76) detected by branch-and-bound method, using the swapping algorithm tree bisection reconnection (TBR) via 100 replications, saving 100 trees per replication.

The exclusion of the dental characters from our phylogenetic analysis produced a 50% majority rule consensus tree in which the relationships between genera are less resolved, as more polytomies are detected (Fig. 9A). This corroborates the hypothesis that inclusion of dental characters in a skeletal-based character matrix is very useful to detect and solve the relationships among batoid taxa (see also Claeson *et al*. 2010; Marramà *et al.*2018d). Moreover, the analysis of the original data matrix using different settings resulted in exactly the same phylogenetic hypothesis (Fig. 9B–D), therefore suggesting that the characters employed here are robust and the resulting systematic arrangement is very stable. Despite the high level of homoplasy, the recognizon of the same phylogenetic hypothesis using different approaches suggests that the data are good, and homoplastic characters can therefore be considered diagnostic for skates (see Carvalho 1996, p. 52).

## Discussion

### Comparative remarks

The affinities of *Ostarriraja parva* gen. et sp. nov. with the skate order Rajiformes *sensu* Naylor *et al*. (2012a) are unquestionably supported herein by the presence of the following features: suprascapulae fused to the median crest of the synarcual forming the pectoral arch, rod-like compound radial articulated with single radial segments in serial fashion, and separated pelvic girdle condyles (e.g. McEachran & Dunn 1998; Lucifora & Vassallo 2002). In this perspective, the similar pelvic girdle morphology of *Ostarriraja* and modern skates may suggest that the peculiar form of benthic locomotion termed ‘punting’ might have been already present in skates since the early Miocene. Another skate character corroborating the inclusion of *Ostarriraja* in Rajiformes is the catenated calcification of the pectoral radials (see Schaefer & Summers 2005). Although this kind of calcification is also present in other batoids (e.g. non-pelagic stingrays), in skates the calcification is unique, being further reduced and consisting of a single chain on the dorsal and ventral sides in all radials (Schaefer & Summers 2005), a character which is unquestionably present also in *Ostarriraja*. Additional features that align the new fossil skate genus with rajiforms, although considered plesiomorphic within Batoidea (Compagno 1977; McEachran *et al.*^1996^; Lucifora & Vassallo 2002), include the lateral expansion of nasal capsules, presence of radials articulating with the scapulocoracoid between meso- and metapterygium, antorbital cartilages not branched, laterally extended and directly joining the propterygia to the nasal capsules, and long and anteriorly directed prepelvic processes of the pelvic girdle.

*Ostarriraja* differs from other skate genera in its unique combination of meristic and dental features (Supplemental material, Table S2). The number of predorsal vertebrae in *Ostarriraja* is low, being probably no more than 70, therefore separating the new fossil genus from *Breviraja*, *Hongeo*, *Leucoraja* and *Malacoraja* and most of the arhynchobatids (80–134). The total number of pectoral radials distinguishes *Ostarriraja* (86) from *Amblyraja*, *Beringraja*, *Breviraja*, *Malacoraja*, *Neoraja, Rajella*, *Brochiraja*, *Pavoraja* and *Pseudoraja* (55–85), and from *Hongeo* and *Irolita* (87–105). Although small, the principal cusp of the teeth in *Ostarriraja* can be considered erect, contrary to being oblique in *Breviraja*, *Malacoraja*, *Atlantoraja*, *Irolita*, *Rhinoraja* and *Rioraja*. The absence of a labial cutting edge is useful to separate *Ostarriraja* from *Amblyraja* and *Rioraja*, whereas the absence of a lingual uvula distinguishes the fossil taxon from *Amblyraja*, *Breviraja*, *Dactylobatus*, *Malacoraja*, *Noeraja*, *Bathyraja*, *Brochiraja* and *Rhinoraja*. The absence of a labial apron separates *Ostarriraja* from *Dipturus*, *Leucoraja* and *Rostroraja*, whereas the high root stem of *Ostarriraja* distinguishes the fossil genus from *Breviraja*, *Dipturus*, *Raja*, *Rajella* and *Rostroraja*, and from *Arhynchobatis*, *Atlantoraja and Pseudoraja.* Finally, *Ostarriraja* can be separated from *Amblyraja*, *Breviraja*, *Dipturus*, *Neoraja*, *Okamejei*, *Raja* and most of the arhynchobatids by the presence of a root coating, and from *Breviraja*, *Malacoraja*, *Arhynchobatis*, *Atlantoraja*, *Bathyraja*, *Irolita* and *Rhinoraja* by having concave mesial and distal cutting edges. Although the selected meristic and dental features are not useful to separate *Ostarriraja* from *Dentiraja*, this living genus can be separated by the presence of narrower and more rectilinear nasal capsules (broad and oval in *Ostarriraja*), and from *Springeria*, *Schroederobatis*, *Cruriraja*, *Sympterygia* and *Psammobatis* by the very different pelvic girdle morphology (see McEachran & Miyake 1990a; McEachran & Dunn 1998).

Dental characters are also useful to separate *Ostarriraja* from other extinct skate genera. The genus *Walteraja* from the Late Cretaceous of Sweden is characterized by having cuspidate teeth in both sexes, female teeth usually longer labio-lingually than they are wide mesio-distally and transverse cutting edges restricted to the top of the cusp (Siverson & Cappetta 2001; Cappetta 2012), whereas in *Ostarriraja* teeth are not cuspidate (at least in females), the crown is longer mesio-distally than labio-lingually, and the transverse cutting edges reach the margins of the crown. Teeth of the Late Cretaceous genus *Mafdetia* are characterized by a crown which is rhombic in occlusal view, having convex transverse cutting edges, a labial cutting edge, and a root which is much narrower than the crown (Werner 1989; Cappetta 2012), whereas in *Ostarriraja* the crown is semi-oval in occlusal view, the cutting edges are concave, the labial cutting edge is absent, and the root is as large as the crown. The fossil genus *Smithraja* from the upper Paleocene–middle Eocene of Europe and Near East (see Cappetta 2012) differs from *Ostarriraja* in having a median concavity on the labial margin of the crown and marginal uvulae, and lacking margino-lingual foramina. Teeth of the genus *Marambioraja* from the Eocene of Antarctica have been described as having a knob-like labial protuberance with a flat oval structure centred on its labial face and cutting edges that do not reach the base of the crown (Engelbrecht *et al.*^2018^), characters which are not present in *Ostarriraja* that, on the contrary, has margino-lingual foramina. The extinct rajid *Mesetaraja* known by a single tooth from the Eocene of Antarctica (Engelbrecht *et al*. 2018) is characterized by having a labial apron, a crown that is wider than the root, a mesio-distally directed narrow cusp and accessory cusplets, a combination of characters, which easily separate it from the new Miocene genus. Finally, *Ostarriraja* can be distinguished from *Oligoraja* from the Oligocene of Germany (see Reinecke 2015) by the different crown shape in occlusal view, a higher root stem, the absence of apron and uvula, and the presence of margino-lingual foramina.

## The fossil record of skates

**Figure 10:**
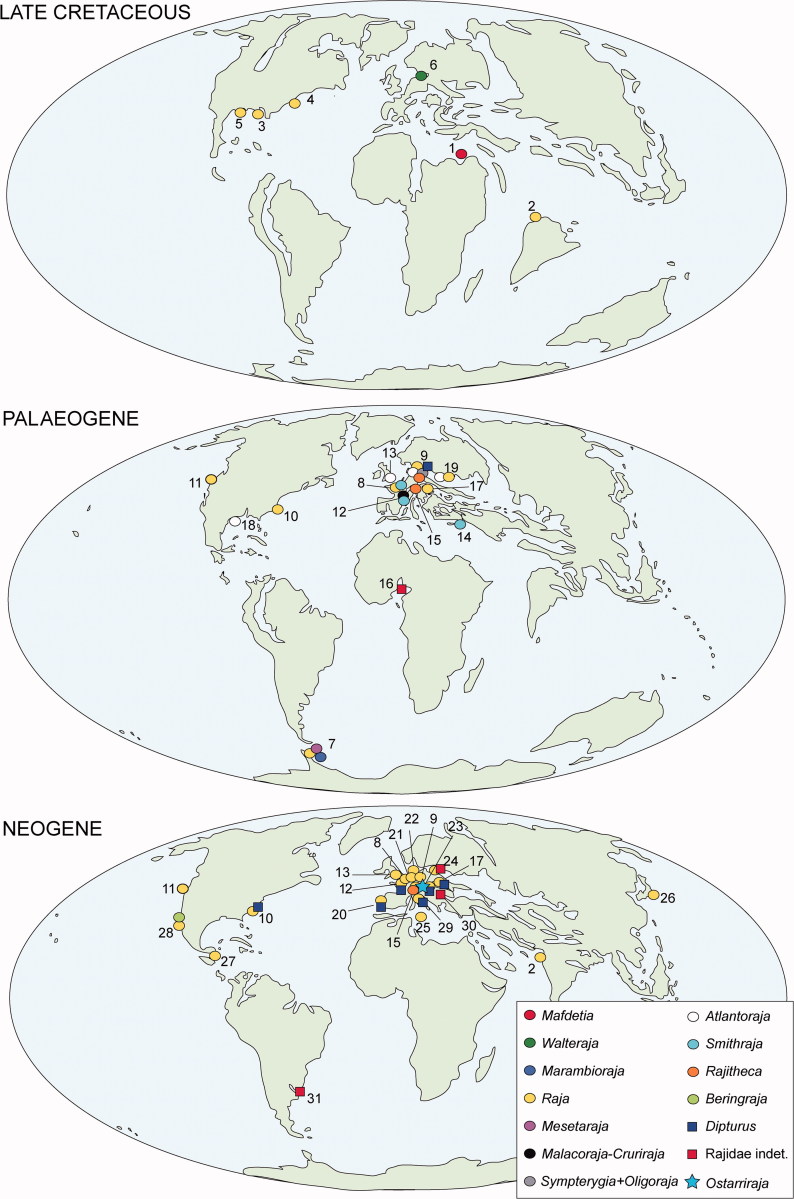
Palaeobiogeography of skates from the Late Cretaceous to Neogene: 1 – Egypt, 2 – India, 3 – Arkansas, 4 – Maryland, 5 – Texas, 6 – Sweden, 7 – Antarctica, 8 – Belgium, 9 – Germany, 10 – South Carolina, 11 – Oregon, 12 – France, 13 – England, 14 – Jordan, 15 – Switzerland, 16 – Niger, 17 – Czech Republic, 18 – Mexico, 19 – Ukraine, 20 – Portugal, 21 – Netherlands, 22 – Denmark, 23 – Austria, 24 – Poland, 25 – Malta, 26 – Japan, 27 – Costa Rica, 28 – California, 29 – Italy, 30 – Slovakia, 31 – Argentina. Data from Fischer-Ooster (1866), Lawley (1876), Leriche (1927), Radwanski (1965), Steininger (1966), Jonet (1968), Ray *et al.* (1968), Cappetta (1970, 2012), Welton (1972), Herman (1974), Langenwalter (1975), Ceuster (1976), Steurbaut & Herman (1978), Glaser (1979), Schultz (1979, 2013), Sahni & Mehrotra (1980), Ward (1984), Itoigawa *et al*. (1985), Werner (1989), Cappetta & Nolf (1991), Prasad & Cappetta (1993), Welton & Farish (1993), Long (1994), Hovestadt & Hovestadt-Euler (1995), Case & Cappetta (1997), Antunes *et al.* (1999), Laurito (1999), Müller (1999), Purdy *et al.* (2001), Siverson & Cappetta (2001), Ward & Bonavia (2001), Müller & Rozenberg (2003), Reinecke *et al*. (2005), Roth & Hoedemakers (2005), Adnet (2006), Becker *et al*. (2006), Cappetta & Cavallo (2006), Sabol & Kovac (2006), Antunes & Balbino (2007), Cahuzac *et al.* (2007), Adnet & Cappetta (2008), González-Barba (2008), Wijnker *et al*. (2008), Brisswalter (2009), Cicimuri & Knight (2009), Schultz *et al*. (2010), Boessenecker (2011, 2013), Cione *et al.* (2012), Reinecke (2015), Pollerspöck & Straube (2017), Engelbrecht *et al.* (2018). Maps are adopted and modified from Scotese (2002).

**Figure 11:**
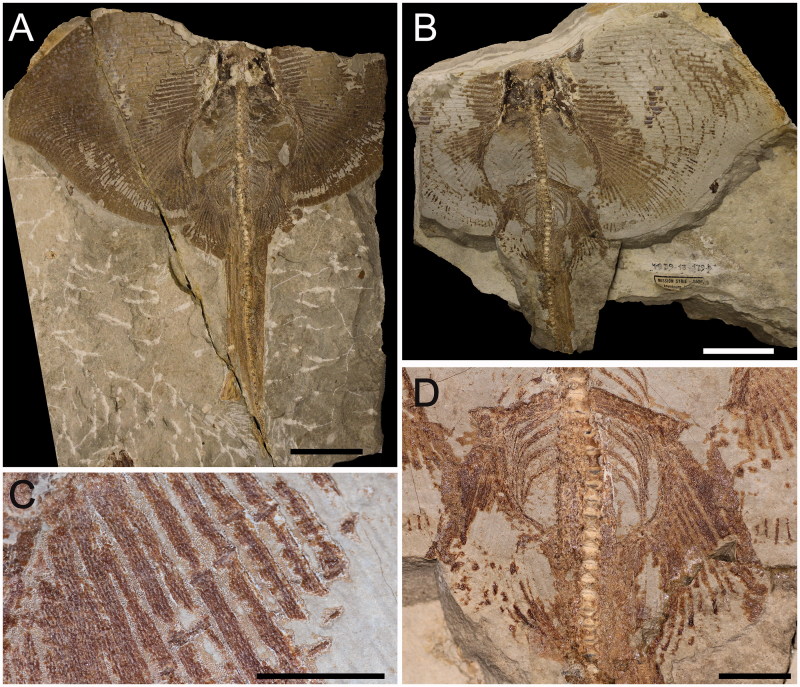
*Rajorhina expansa* (Davis, 1887) from the Late Cretaceous (Cenomanian) of Haqel, Lebanon. **A,** MNHN HAK558; **B,** MNHN HAK561, counterpart; **C,** close-up of the pectoral radials showing the crustal calcification; **D,** detail of the pelvic fins. Scale bars: A, B = 20 mm; C, D = 10 mm.

Although skates represent today the most diverse group of batoid fishes, their fossil record is quite scarce compared to that of other rays and heavily biased towards isolated teeth (Fig. 10). Some of the oldest putative skates such as *Rajorhina expansa* (Davis, 1887) and three species of *Cyclobatis* Egerton, 1844 (*C*. *major*, *C. oligodactylus*, *C. tuberculatus*) that are reported from the Late Cretaceous (Cenomanian) deposits of Haqel and Hjoula in Lebanon (Cappetta 1980) are represented by articulated skeletons. However, the affinities of these taxa with the true skates are unclear and doubtful, since they lack most of the diagnostic features of Rajiformes (Siverson & Cappetta 2001; Cappetta 2012). The two known specimens of *Rajorhina expansa* (*Pararaja* in Cappetta 1980) are incomplete and lack the rostral region, and their alignment with skates was only based on the similar outline of the pectoral fins, presence of long claspers, and presence of large thorns on discs (Cappetta 1980). One of the two specimens, MNHN HAK558-561 in part and counterpart, was examined for the present study (Fig. 11). The pelvic fins of *R. expansa* (Fig. 11D) are clearly not bilobed; the compound radial and basipterygium articulate with pelvic girdle condyles which are close together, not distinctly separated, and do not allow for the articulation of pelvic radials directly with the pelvic girdle between the two condyles as in living skates (McEachran & Dunn 1998; Lucifora & Vassallo 2002). In *Rajorhina* there are no long anteriorly directed prepelvic processes but small lateral processes directed towards the exterior (see also Cappetta 1980, 2012). Both individuals of *R. expansa* have long claspers, but they do not show apparently the malar or alar thorns typical of male skates (see also Cappetta 1980). The pectoral radials of *Rajorhina* are entirely covered by small tesserae (Fig. 11C), typical of the ‘crustal’ calcification of guitarfishes, thornbacks, sawfishes, electric rays and pelagic stingrays (Schaefer & Summers 2005), very different from the reduced ‘catenated’ calcification of *Ostarriraja* and modern skates. Moreover, teeth of *Rajorhina* were described as of anaulacorhizid type with a broad and flat root (Cappetta 1980), whereas teeth of the skates mostly show holaulacorhizy or secondary hemiaulacorhizy (Herman *et al.*^1994^, ^1995^, ^1996^). Finally, the presence of ribs in *Rajorhina* (Fig. 11D) aligns this taxon more with Rhinopristiformes than with skates. The absence of ribs has been suggested to be a synapomorphic character of stingrays of the order Myliobatiformes (e.g. Carvalho *et al*. 2004; Aschliman *et al*. 2012a). However, our examination of the comparative material and the radiographs in the available literature detected their absence also in skates. From this perspective, since no reliable diagnostic features of skates appear to be present in *R. expansa*, the presence of scapular thorns reported in Cappetta (1980), a similar pectoral disc shape and long claspers might represent homoplastic characters achieved independently in *Rajorhina* and modern skates.

**Figure 12:**
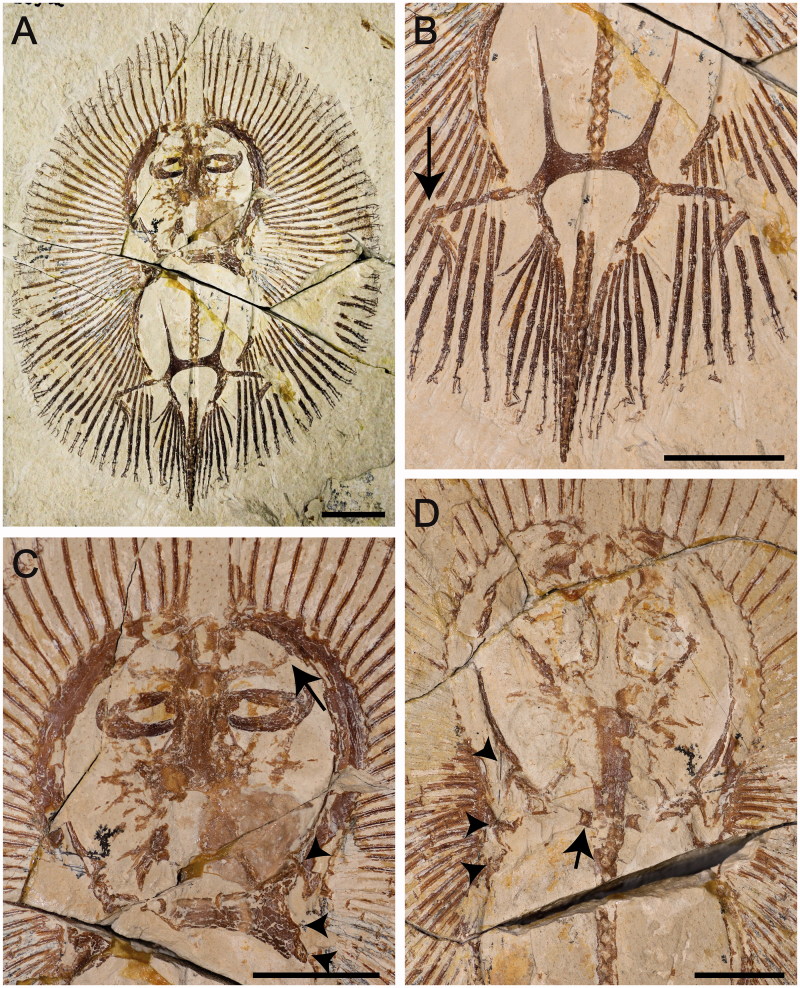
*Cyclobatis oligodactylus* Egerton, 1844 from the Late Cretaceous (Cenomanian) of Hjoula, Lebanon. **A,** MNHN F.HDJ505a; **B,** detail of the pelvic girdle and fins, showing the compound radial articulating with several radials in parallel fashion (arrow); **C,** detail of the head, showing the articulation of nasal capsules with propterygia (arrow); **D,** MNHN F.HDJ505b, counterpart, detail of the of the pectoral girdle showing the suprascapulae (arrow). The arrowheads in C and D show the distance between pro-, meso- and metacondyles. Scale bars: 10 mm.

Most authors have assigned *Cyclobatis* to the skates (e.g. Egerton 1844; Goodrich 1909; Dechaseaux 1937; Cappetta 1980, 2012). A specimen of *C. oligodactylus* Egerton, 1844 (MNHN F.HDJ504, MNHN F.HDJ505, in part and counterpart) and one of *C. major* Davis, 1887 (MNHN HAK555) were examined for the present study (Fig. 12). Although the pelvic girdle of *Cyclobatis* shows long, anteriorly directed prepelvic processes, and pelvic fins that seem to be differentiated into anterior and posterior lobes with a large gap of radials between the compound radial and the basipterygium, resembling the condition of the skate genera *Cruriraja* and *Anacanthobatis*, the compound radial of *Cyclobatis* articulates with several radial segments in a parallel fashion (Fig. 12B; see also Cappetta 1980, fig. 25) contrary to the condition of skates in which the compound radial articulates with single radial segments in a serial fashion (Fig. 4C; see also Holst & Bone 1993, fig. 1; Lucifora & Vassallo 2002, fig. 2; Aschliman *et al.*2012a). Moreover, the morphology of the cranial skeleton of *Cyclobatis* (Fig. 12C) is unique and very different from that of any other extant batoid in the shape of the rostral cartilage and nasal capsules, these latter contacting directly the propterygium because of the apparent absence of antorbital cartilages (Cappetta 1980, 2012). As in most of the batoids, the suprascapulae of *Cyclobatis* form a small, narrow and straight bar, which articulates with the neural arches of the vertebrae just posterior to the synarcual (Fig. 12D; see also Cappetta 1980, fig. 24) as in sawfishes and electric rays (McEachran *et al.*^1996^), whereas skates are unique among batoids in that the suprascapulae are fused to the median crest of the synarcual forming the pectoral arch (Garman 1913; Claeson 2008, 2011; Aschliman *et al.*2012a). The pectoral radials of *Cyclobatis* are covered at least in their proximal part by small tesserae, typical of the ‘crustal’ calcification, very different from the reduced catenated calcification of *Ostarriraja* and modern skates (Schaefer & Summers 2005). Finally, pro-, meso- and metacondyles of the scapulocoracoid are about at the same distance, or the scapulocoracoid is elongated between the mesocondyle and the metacondyle in skates (Nishida 1990, fig. 32; McEachran *et al*. 1996, fig. 9; McEachran & Dunn 1998). *Cyclobatis*, on the contrary, shows a condition similar to that of myliobatiforms (see also Nishida 1990, figs 30–32; Lovejoy 1996, fig. 9; McEachran *et al.*^1996^, fig. 9), in which the scapulocoracoid is elongated between the pro- and mesocondyle (Fig. 12C, D). These and other differences in tooth morphology (see Siverson & Cappetta 2001) highlight the importance of detailed revisions of *Cyclobatis*, as well as most of the batoids from the Late Cretaceous of Lebanon.

Another batoid represented by partial skeletons and included within skates by Cappetta (1980) is *Raja*? *davisi* (Fowler, 1958) from the upper Santonian of Sahel Aalma, Lebanon. However, the impossibility of recognizing any skate character because of the poor preservation of the material and the lack of teeth in these specimens makes any assignment of the species to Rajiformes impossible to establish (Siverson & Cappetta 2001; Cappetta 2012). From this perspective, since no reliable taxa represented by holomorphic specimens can be confidentially assigned to skates, *Ostarriraja parva* is considered here to be the first unquestionable skate represented by a partially complete and articulated skeleton.

The oldest true representative of skates can be considered *Mafdetia* Werner, 1989, represented by isolated teeth from the Cenomanian of Egypt (see also Fig. 10), although its inclusion in the family Rajidae is questionable according to Cappetta (2012) who prefers to consider the taxon as Rajoidei *incertae familiae*. Although skates have been reported from the Maastrichtian of India as *Raja sudhakari*, and the United States (Arkansas, Maryland and Texas) as *R. farishi* (see Glaser 1979; Prasad & Cappetta 1993; Welton & Farish 1993; Case & Cappetta 1997; Becker *et al.*^2006^; Cappetta 2012), the earliest known skate with modern tooth morphology comparable to living taxa might be *Walteraja exigua* from the Maastrichtian of southern Sweden (Siverson & Cappetta 2001). Isolated teeth of skates are more common in Cenozoic strata and in particular in the Neogene, in accordance with the diversification time estimates for the lineage (e.g. Bertozzi *et al.*^2016^). In the Palaeogene, the most common taxon appears to be *Raja*, although several fossil teeth with ‘rajoid’ morphology included traditionally in this wastebasket genus probably belong to different genera (Cappetta 2012). However, unambiguous occurrences of *Raja* have been recently reported from the Eocene of Antarctica, along with the new genera *Marambioraja* and *Mesetaraja* (Long 1994; Engelbrecht *et al.*^2018^), and Oregon (Welton 1972), and the Oligocene of Belgium, Czech Republic, Germany, Ukraine, Oregon and South Carolina (Welton 1972; Steurbaut & Herman 1978; Hovestadt & Hovestadt-Euler 1995; Müller & Rozenberg 2003; Reinecke *et al.*^2005^; Cicimuri & Knight 2009; Cappetta 2012; Reinecke 2015). In the Neogene, *Raja* teeth have been reported in the Miocene of Austria, Czech Republic, Denmark, France, Germany, Malta, Netherlands, Poland, Portugal, Switzerland, California, Costa Rica, Oregon, India and Japan (Leriche 1927; Radwanski 1965; Cappetta 1970, 2012; Welton 1972; Sahni & Mehrotra 1981; Itoigawa *et al.*^1985^; Antunes *et al.*^1999^; Laurito 1999; Ward & Bonavia 2001; Roth & Hoedemakers 2005; Antunes & Balbino 2007; Wijnker *et al.*^2008^; Schultz *et al*. 2010; Boessenecker 2011; Schultz 2013; Pollerspöck & Straube 2017), whereas the genus has been reported from Pliocene deposits in Belgium, England, France, The Netherlands, Italy, California, and North and South Carolina (Lawley 1876; Herman 1974; Ceuster 1976; Cappetta & Nolf 1991; Purdy *et al.*^2001^; Wijnker *et al.*^2008^; Boessenecker 2011; Cappetta 2012), and from the Pleistocene of California, Oregon and Virginia (Ray *et al.*^1968^; Langenwalter 1975; Boessenecker 2013).

Teeth from the early to middle Eocene of southern France, assigned to *Raja* by Adnet (2006) and Cahuzac *et al*. (2007), have been attributed to the morphological group *Malacoraja*-*Cruriraja* by Cappetta (2012); those from the Ypresian London Clay included in *Raja* by Ward (1984) have been regarded as similar to the modern genus *Atlantoraja* by Siverson & Cappetta (2001); *Atlantoraja* should be also present in the Oligocene of Mexico (as *R*. aff. *heinzelini* in González-Barba 2008) and Ukraine (Müller & Rozenberg 2003); some of the teeth from the Oligocene of northern Germany assigned to *Raja* by Steurbaut & Herman (1978) have been recently included in the extant genera *Atlantoraja* and *Dipturus* by Reinecke (2015) who recognized also the presence of *Sympterygia* and the new fossil genus *Oligoraja* in the same deposit; some teeth from the Miocene to the Pleistocene of California identified as *R*. cf. *binoculata* should be referred to *Beringraja binoculata* (see Boessenecker 2011). The extant genus *Dipturus* was reported from the Miocene of Austria, Czech Republic, France and Portugal (Jonet 1968; Antunes *et al.*^1999^; Brisswalter 2009; Schultz *et al.*^2010^; Cappetta 2012; Schultz 2013), and the Pliocene of France, Italy and North Carolina (Cappetta & Nolf 1991; Müller 1999; Cappetta & Cavallo 2006; Cappetta 2012). *Smithraja*, which also is based on isolated teeth, occurs in the upper Paleocene of the Near East to the middle Eocene of France and Belgium (Adnet 2006; Adnet & Cappetta 2008; Cappetta 2012). Another genus, *Rajitheca* Steininger, 1966, is only represented by egg cases from the Oligocene of Switzerland and Germany and the Miocene of Switzerland (Fischer-Ooster 1866; Steininger 1966). Finally, undetermined rajid teeth have been reported from the upper Paleocene of Niger (Cappetta 2012), and the Miocene of Argentina, Germany, Poland and Slovakia (Schultz 1979; Sabol & Kovac 2006; Cione *et al*. 2012; Pollerspöck & Straube 2017).

Contrary to McEachran & Miyake (1990b) who proposed a Pacific origin for skates, the distribution of their fossil record appears to be more consistent with the dispersal scenario hypothesized by Long (1994), who suggested that skates evolved in the western Tethys and North Boreal seas in the Late Cretaceous–early Palaeogene and emigrated towards the Southern Hemisphere and Antarctica during the early–middle Eocene across a dispersal corridor along the continental margins of the western Atlantic Ocean, a trend also detected for other elasmobranchs (e.g. the extinct sand tiger shark *Brachycarcharias*; see Marramà *et al.*2018b). On the contrary, the invasion of the Pacific Ocean by skates could have occurred through the Arctic Ocean, or through the Panamic Seaway, or from Antarctica (Long 1994). It is noteworthy that no fossil skates have been reported from Indo-Pacific areas so far, suggesting that the current limited distribution of the few species in these regions (see Last *et al*. 2016) might have occurred only recently. Molecular estimates of divergence times indicate a recent origin and rapid dispersal of the present species in the Mediterranean Sea and Eastern Atlantic from the middle Miocene to the Pleistocene, related to climatic and geological events in the Mediterranean area (Valsecchi *et al.*^2005^). From this perspective it is likely that modern species in the Mediterranean Sea and Eastern Atlantic originated from, or at least co-existed with, early Miocene taxa, possibly including *Ostarriraja*, in the Central Paratethys.

## Conclusions

A new fossil representative of the skates, *Ostarriraja parva* gen. et sp. nov., is recognized and described from early Miocene strata of Upper Austria, which were part of the Central Paratethys during the middle Burdigalian. The new taxon, represented by a partially complete and articulated skeleton, represents the first unquestionable skeletal record of skates, since a comparison with putative holomorphic skates from the Late Cretaceous suggests that the latter cannot be ascribed to Rajiformes *sensu* Naylor *et al*. (2012a), highlighting the importance of deep anatomical investigation for the Late Cretaceous batoid taxa. The phylogenetic analysis recovered *Ostarriraja* as the basal-most skate and a dichotomous nature of the relationships of the living rajiforms, in accordance with previous morphological studies. The analysis of the fossil record of skates seems to corroborate the hypothesis that these batoids evolved in the western Tethys and North Boreal seas in the Late Cretaceous–early Palaeogene, and that the present distribution of the species in Mediterranean and Eastern Atlantic is the result of the climatic and geological events that occurred in the Central Paratethys and Mediterranean area in the Miocene.

## Supplementary Material

Supplemental_Appendix_B.docx

Supplemental_Appendix_A.docx

Supplemental_Material_Tables_S1_and_S2.docx

## References

[CIT0001] Adnet S. 2006 Nouvelles faunes de Sélaciens (Elasmobranchii, Neoselachii) de l’Eocène moyen des Landes (Sud-Ouest, France). Implication dans la connaissance des communautés de sélaciens d’eaux profondes. Palaeoichthyologica, 10, 1–128.

[CIT0002] Adnet S. & Cappetta H. 2008 New fossil Triakidae (Chondrichthyes, Carcharhiniformes) from the Upper Ypresian of Prémontré (Aisne, France) with comments on fossil record of the family. Acta Palaeontologica Polonica, 53, 433–448.

[CIT0003] Allen P. A., Mange-Rajetzky M., Matter A. & Homewood P. 1985 Dynamic palaeogeography of open Burdigalian sea-way, Swiss Molasse Basin. Eclogae Geologicae Helvetiae, 79, 351–381.

[CIT0004] Antunes M. T. & Balbino A. C. 2007 Rajiformes (Neoselachii, Batomorphii) from the Alvalade basin, Portugal. Annales de *Paleontologie*, 93, 107–119.

[CIT0005] Antunes M. T., Balbino A. C. & Cappetta H. 1999 Selaciens du Miocene terminal du Bassin d’ Alvalade (Portugal). Essai de synthese. Ciencias da Terra, 13, 115–129.

[CIT0006] Aschliman N. C., Claeson K. M. & McEachran J. D. 2012a Phylogeny of Batoidea. Pp. 57–96 in Carrier J. C., Musick J. A. & Heithaus M. R. (eds) *Biology of sharks**and**their relatives* 2nd edition CRC Press, Boca Raton.

[CIT0007] Aschliman N. C., Nishida M., Miya M., Inoue J. G., Rosana K. M. & Naylor G. J. P. 2012b Body plan convergence in the evolution of skates and rays (Chondrichthyes: Batoidea). Molecular Phylogenetics and Evolution, 3, 28–42. 10.1016/j.ympev.2011.12.012 22209858

[CIT0008] Bachmann A. 1973 Die Silicoflagellaten aus dem Stratotypus des Ottnangien. Pp. 275–295 in Papp A., Rögl & F. Senes J. (eds) *M2 Ottnangien. Die Innviertler, Salgótarjáner, Bántapusztaer Schichtengruppe und die Rzehakia Formation. Chronostratigraphie und Neostratotypen, Miozän der Zentralen Paratethys*. Verlag der Slowakischen Akademie der Wissenschaften, Bratislava.

[CIT0009] Becker M. A., Chamberlain J. A. & Wolf G. E. 2006 Chondrichthyans from the Arkadelphia Formation (Upper Cretaceous: upper Maastrichtian) of Hot Spring County, Arkansas. Journal of Paleontology, 80, 700–716.

[CIT0010] Berg L. S. 1937 A classification of fish-like vertebrates. Bulletin de l’Académie des Sciences de l’URSS. Classe des Sciences Mathématiques et Naturelles, 1937, 1277–1280.

[CIT0011] Berggren W. A., Kent D. V., Swisher C. C. III & Aubry M.-P. 1995 A revised Cenozoic geochronology and chronostratigraphy. *Society for Sedimentary Geology*, Special Publication, 54, 129–212.

[CIT0012] Bertozzi T., Lee M. S. Y. & Donnellan S. C. 2016 Stingray diversification across the end-Cretaceous extinctions. Memoirs of Museum Victoria, 74, 379–390.

[CIT0013] Bigelow H. B. & Schroeder S. C. 1948 New genera and species of batoid fishes. Journal of Marine Research, 7, 543–566.

[CIT0014] Boessenecker R. W. 2011 A new marine vertebrate assemblage from the Late Neogene Purisima Formation in Central California, Part I: Fossil sharks, bony fish, birds, and implications for the age of the Purisimia Formation west of the San Gregorio Fault. PalArch’s Journal of Vertebrate Palaeontology, 8(4), 1–30.

[CIT0015] Boessenecker R. W. 2013 Taphonomic implications of barnacle encrusted sea lion bones from the Middle Pleistocene Port Orford Formation, coastal Oregon. Journal of Paleontology, 87, 657–663.

[CIT0018] Brisswalter G. 2009 Inventaire des elasmobranches (requins, raies, chimeres) des depots molassiques du Sud-Luberon (Miocenesuperieur). Courriers scientifiques du Parc Régional du Lubéron, *Hors Série*, 1–100.

[CIT0019] Brzobohatý R. & Schultz O. 1973 Die Fischfauna der Innviertler Schichtengruppe und der Rzehakia Formation. Pp. 652–693 in Papp A., Rögl & F. Senes J. (eds) M2 Ottnangien. Die Innviertler, Salgótarjáner, Bántapusztaer Schichtengruppe und die Rzehakia Formation. Chronostratigraphie und Neostratotypen, Miozän der Zentralen Paratethys. Verlag der Slowakischen Akademie der Wissenschaften, Bratislava.

[CIT0020] Cahuzac B., Adnet S., Cappetta H. & Vullo R. 2007 The fossil species and genera of Selachians fishes (Cretaceous, Tertiary) created in the Aquitaine Basin; inventory, taxonomics. Bulletin de la Société Linnéenne de Bordeaux, 142(35), 3–43.

[CIT0021] Cappetta H. 1970 Les selaciens du Miocene de la region de Montpellier. Palaeovertebrata, 1970, 1–139.

[CIT0022] Cappetta H. 1980 Les selaciens du Cretace superieur du Liban. II: Batoides. Palaeontographica Abteilung A, 168, 149–229.

[CIT0023] Cappetta H. 2012 Handbook of Paleoichthyology – Chondrichthyes – Mesozoic and Cenozoic Elasmobranchii: teeth. Verlag Dr. Friedrich Pfeil, Munich.

[CIT0024] Cappetta H. & Cavallo O. 2006 Les selaciens du Pliocene de la region d’Alba (Piémont, Italie NordOuest). Rivista Piemontese di Storia Naturale, 27, 33–76.

[CIT0025] Cappetta H. & Nolf D. 1991 Les sélaciens du Pliocène inférieur de Le-Puget-sur-Argens (Sud-Est de la France). Palaeontographica Abteilung A, 218, 49–67.

[CIT0026] Carvalho M. R. 1996 Higher-level elasmobranch phylogeny, basal squaleans, and paraphyly. Pp. 35–62 in M. J. Stiassny, L. R. Parenti & G. D. Johnson (eds) *Interrelationships of fishes* Academic Press, London.

[CIT0028] Carvalho M. R., Maisey J. C. & Grande L. 2004 Freshwater stingrays of the Green River Formation of Wyoming (Early Eocene), with the description of a new genus and species and an analysis of its phylogenetic relationships (Chondrichthyes: Myliobatiformes). Bulletin of the American Museum of Natural History, 284, 1–136.

[CIT0029] Case G. R. & Cappetta H. 1997 A new selachian fauna from the Late Maastrichtian of Texas (Upper Cretaceous/Navarroan; Kemp Formation). Münchner Geowissenschaftliche Abhandlungen (A: Geologie und Paläontologie), 34, 131–189.

[CIT0030] Ceuster J. 1976 Stratigrafische interpretatie van jong cenozoische afzeettingen bij Rumst (Belgie, Provincie Antwerpen) en beschrijving van de in een post-Mioceen basisgrind aangetrofen vissenfauna. II. Systematische beschrijvingen en conclusies. Mededelingen van de Werkgroep voor Tertiaire en Kwartaire Geologie, 13(4), 119–172.

[CIT0031] Cicimurri D. J. & Knight J. L. 2009 Late Oligocene sharks and rays from the Chandler Bridge Formation, Dorchester County, South Carolina, USA. Acta Palaeontologica Polonica, 54, 627–647.

[CIT0032] Cione A. L., Cabrera D. A & Barla M. J. 2012 Oldest record of the Great White Shark (Lamnidae, Carcharodon; Miocene) in the Southern Atlantic. Geobios, 45, 167–172.

[CIT0033] Claeson K. M. 2008 Variation of the synarcual in the California Ray, *Raja inornata* (Elasmobranchii: Rajidae). Acta Geologica Polonica, 58, 121–126.

[CIT0034] Claeson K. M. 2011 The synarcual cartilage of batoids with emphasis on the synarcual of Rajidae. Journal of Morphology, 272, 1444–1463. 21780157 10.1002/jmor.10996

[CIT0035] Claeson K. M., O’Leary M. A., Roberts E. M., Sissoko F., Bouaré M., Tapanila L., Goodwin D. & Gottfried M. D. 2010 First Mesozoic record of the stingray *Myliobatis wurnoensis* from Mali and a phylogenetic analysis of Myliobatidae incorporating dental characters. Acta Palaeontologica Polonica, 55, 655–674.

[CIT0036] Compagno L. J. V. 1977 Phyletic relationships of living sharks and rays. American Zoologist, 17, 303–322.

[CIT0037] Compagno L. J. V. 1999 Endoskeleton. Pp. 69–92 in Hamlett W. C. (ed.) Sharks, skates, and rays: the biology of elasmobranch fishes. Johns Hopkins University Press, Baltimore.

[CIT0038] Davis J. W. 1887 The fossil fishes of the chalk of Mount Lebanon, in Syria. Scientific Transactions of the Royal Dublin Society, 3, 457–636.

[CIT0039] Dean M. N. & Summers A. P. 2006 Mineralized cartilage in the skeleton of chondrichthyan fishes. Zoology, 109, 164–168. 16584875 10.1016/j.zool.2006.03.002

[CIT0040] Dechaseaux C. 1937 Sur le genre *Cyclobatis* et sa position systématique parmi l’ensemble des sélaciens. *Notes et Memoires, Haut Commissariat de la Republique francaise en Syrie et au Liban*, 2, 201–206.

[CIT0041] Egerton P. M. G. 1844 Description of a fossil ray from Mount Lebanon (*Cyclobatis oligodactylus*). Proceedings of the Geological Society of London, 4, 442–446.

[CIT0042] Engelbrecht A., Mörs T., Reguero M. A. & Kriwet J. 2018 Skates and rays (Elasmobranchii, Batomorphii) from the Eocene La Meseta and Submeseta formations, Seymour Island, Antarctica. Historical Biology, doi:10.1080/08912963.2017.1417403 10.1080/08912963.2017.1417403 PMC6650296 31337928

[CIT0043] Fischer-Ooster C. 1866 Palaontologische Mitteilungen. 2. Ueber fossile Seemause. Mittheilungen der Naturforschenden Gesellschaft in Bern, 1865, 267–268.

[CIT0044] Fowler H. W. 1958 Some new taxonomic names of fishlike vertebrates. Notulae Naturae of the Academia of Natural Sciences of Philadelphia, 310, 1–16.

[CIT0045] Garman S. 1913 The Plagiostoma (sharks, skates, and rays). Memoirs of the Museum of Comparative Zoology of Harvard College, 36, 1–528.

[CIT0046] Glaser J. D. 1979 Collecting fossils in Maryland. Maryland Geological Survey Educational Series, 4, 1–83.

[CIT0047] Goloboff P. A., Farris J. S. & Nixon K. C. 2008 TNT, a free program for phylogenetic analysis. Cladistics, 24, 774–786.

[CIT0048] González-Barba G. 2008 *Descripción de la asociación faunistica de elasmobranquios fósiles del conglomerado basa en las formaciones San Gregorio y el Cien (Oligoceno Temprano) de Baja California Sur, Mexico*. Unpublished PhD thesis, Centro Interdisciplinario de Ciencias Marinas, 226 pp.

[CIT0049] Goodrich E. S. 1909 Vertebrata Craniata. I. Cyclostomes and fishes. Pp. 1–518 in Lankester E. R. (ed.) A Treatise on Zoology. Adam A. Charles Black, London.

[CIT0050] Gravendeel R., Van Neer W. & Brinkhuizen D. 2002 An identification key for dermal denticles of Rajidae from the North Sea. International Journal of Osteoarchaeology, 12, 420–441.

[CIT0051] Grunert P., Soliman A., Ćorić S., Scholger R., Harzhauser M. & Piller W. E. 2010a Stratigraphic re-evaluation of the stratotype for the regional Ottnangian stage (Central Paratethys, middle Burdigalian). Newsletter on Stratigraphy, 44, 1–16.

[CIT0052] Grunert P., Soliman A., Harzhauser M., Müllegger S., Piller W. E., Roetzel R. & Rögl F. 2010b Upwelling conditions in the Early Miocene Central Paratethys Sea. Geologica Carpathica, 61(2), 129–145.

[CIT0053] Herman J. 1974 Quelques restes de selaciens recoltes dans les sables du Kattendijk et Kallo. 1 – Selachii, Euselachii. Bulletin de la Societe beige de Geologie, 83(1), 15–31.

[CIT0054] Herman J., Hovestadt-Euler M., Hovestadt D. C. & Stehmann M. 1994 Contributions to the study of the comparative morphology of teeth and other relevant ichthyodorulites in living supra-specific taxa of Chondrichthyan fishes. Part B: Batomorphii No. 1a: Order Rajiformes – Suborder Rajoidei – Family: Rajidae Genera and Subgenera: *Anacanthobatis* (*Schroederobatis*), *Anacanthobatis* (*Springeria*), *Breviraja*, *Dactylobatus*, *Gurgesiella* (*Gurgesiella*), *Gurgesiella* (*Fenestraja*), *Malacoraja*, *Neoraja* and *Pavoraja* *Bulletin de l’Institut Royal des Sciences Naturelles de Belgique, Biologie*, 64, 165–207.

[CIT0055] Herman J., Hovestadt-Euler M., Hovestadt D. C. & Stehmann M. 1995 Contributions to the study of the comparative morphology of teeth and other relevant ichthyodorulites in living supra-specific taxa of Chondrichthyan fishes. Part B: Batomorphii No. 1b: Order Rajiformes – Suborder Rajoidei – Family: Rajidae – Genera and Subgenera: *Bathyraja* (with a deep-water, shallow-water and transitional morphotype), *Psammobatis*, *Raja* (*Amblyraja*), *Raja* (*Dipturus*), *Raja* (*Leucoraja*), *Raja* (*Raja*), *Raja* (*Rajella*) (with two morphotypes), *Raja* (*Rioraja*), *Raja* (*Rostroraja*), *Raja lintea*, and *Sympterygia. Bulletin de l’Institut Royal des Sciences Naturelles de Belgique, Biologie*, 65, 237–307.

[CIT0056] Herman J., Hovestadt-Euler M., Hovestadt D. C. & Stehmann M. 1996 Contributions to the study of the comparative morphology of teeth and other relevant ichthyodorulites in living supra-specific taxa of Chondrichthyan fishes. Part B: Batomorphii No. 1c: Order Rajiformes – Sub order Rajoidei – Family: Rajidae – Genera and Subgenera: *Arhynchobatis*, *Bathyraja richardsoni*-type, *Cruriraja*, *Irolita*, *Notoraja*, *Pavoraja* (*Insentiraja*), *Pavoraja* (*Pavoraja*), *Pseudoraja*, *Raja* (*Atlantoraja*), *Raja* (*Okamejei*) and *Rhinoraja* *Bulletin de l’Institut Royal des Sciences Naturelles de Belgique, Biologie*,66, 179–236.

[CIT0057] Holst R. J. & Bone Q. 1993 On bipedalism in skates and rays. Philosophical Transactions of the Royal Society of *London, B*, 339, 105–108.

[CIT0058] Hovestadt D. C. & Hovestadt-Euler M. 1995 Additions to the fauna of the Boom Clay Formation (Rupelian, Oligocene). Taxonomic adjustments on the Scyliorhinidae and Rajoidae, discovery of a dasyatid species (Pisces, Chondrichthyes) and of a curculionid species (Insecta, Coleoptera). Belgian Geological Survey, Professional Paper: Elasmobranches et Stratigraphie, 278, 261–282.

[CIT0059] Hovestadt D., Hovestadt-Euler M. & Micklich N. 2010 A review of the chondrichthyan fauna of Grube Unterfeld (Frauenweiler) clay pit. Kaupia, 17, 57–71.

[CIT0060] Hulley P. A. 1973 Interrelationships within the Anacanthobatidae (Chondrichthyes, Rajoidea), with a description of the lectotype of *Anacanthobatis marmoratus* von Bonde & Swart, 1923. Annals of the South African Museum, 62(4),131–158.

[CIT0061] Huxley T. H. 1880 On the application of the laws of evolution to the arrangement of the Vertebrata, and more particularly of the Mammalia. *Proceedings of the Zoological Society*, London, 43, 649–662.

[CIT0062] Itoigawa J., Nishimoto H., Karasawa H. & Okumura Y. 1985 Miocene fossils of the Mizunami Group, central Japan. 3. Elasmobranchs. Monograph of the Mizunami Fossil Museum, 5, 1–89.

[CIT0064] Jeong C.-H. & Nakabo T. 2009 *Hongeo*, a new skate genus (Chondrichthyes: Rajidae), with redescription of the type species. Ichthyological Research, 56, 140–155.

[CIT0065] Jonet S. 1968 Notes d’ichthyologie miocene portugaise. V. – Quelques batoldes. Revista da Faculdade de Ciências, Universidade de Lisboa, 15(2), 233–258.

[CIT0066] Koester D. M. 2003 Anatomy and motor pathways of the electric organ of skates. The Anatomical Record Part A, 273(A), 648–662. 10.1002/ar.a.10076 12808649

[CIT0067] Koester D. M. & Spirito C. P. 2003 Punting: an unusual mode of locomotion in the little skate, *Leucoraja erinacea* (Chondrichthyes: Rajidae). Copeia, 2003, 553–561.

[CIT0068] Langenwalter P. E. 1975 Chordates: the fossil vertebrates of the Los Angeles-Long Beach Harbor region. Pp. 36–54 in Soule D. F. & Oguri M. (eds) Marine studies of San Pedro Bay, California. University of Southern California, Los Angeles.

[CIT0070] **Last,** P. R. & Compagno L. J. V. 1999 Order Rajiformes. Pp. 1452–1466 in Carpenter K. E. & Niem V. H. (eds) *FAO species identification guide for fishery purposes. The living marine resources of the Western Central Pacific. Volume 3. Batoid fishes, chimaeras and bony fishes, part 1 (Elopidae to Linophrynidae).* FAO, Rome.

[CIT0074] Last P. R., White W., Carvalho M. R., Séret B., Stehmann M. & Naylor G. 2016 Rays of the world. CSIRO Publishing, Clayton North, 790 pp.

[CIT0075] Laurito C. A. 1999 Los selaceos fósiles de la localidad de Alto Guayacan (y otros ictiolitos asociados): Mioceno Superior-Plioceno Inferior de la formación Uscari, provincia de Limón, Costa Rica. Laurito. C.A., 168 pp.

[CIT0076] Lawley R. 1876 Nuovi studi sopra ai pesci ed altri vertebrati fossili delle colline Toscane. Tipografia dell’Arte della Stampa, Florence, 122 pp.

[CIT0077] Leriche M. 1927 Les poissons de la Molasse suisse. Mémoires de la Société paléontologique Suisse, 46(1), 1–55.

[CIT0078] Long D. J. 1994 Quarternary colonalization or Paleogene persistence? Historical biogeography of skates (Chondrichthyes: Rajidae) in the Antarctic Ichthyofauna. Paleobiology, 20, 215–228.

[CIT0079] Lovejoy N. R. 1996 Systematics of myliobatoid elasmobranchs: with emphasis on the phylogeny and historical biogeography of neotropical freshwater stingrays (Potamotrygonidae: Rajiformes). Zoological Journal of the Linnean Society, 117, 207–257.

[CIT0080] Lucifora L. O. & Vassallo A. I. 2002 Walking in skates (Chondrichthyes, Rajidae): anatomy, behaviour and analogies to tetrapod locomotion. Biological Journal of the Linnean Society, 77, 35–41.

[CIT0081] Macesic L. J. & Kajiura S. M. 2010 Comparative punting kinematics and pelvic fin musculature of benthic batoids. Journal of Morphology, 271, 1219–1228. 20623523 10.1002/jmor.10865

[CIT0082] Maddison W. P. & Maddison D. R. 2008 Mesquite: a modular system for evolutionary analysis. Version 3 03 Updated at: http://mesquiteproject.org, accessed 9 April 2018.

[CIT0083] Marramà G., Claeson M. K., Carnevale G. & Kriwet J. 2018d Revision of Eocene electric rays (Torpediniformes, Batomorphii) from the Bolca Konservat-Lagerstätte, Italy, reveals the first fossil embryo in situ in marine batoids and provides new insights into the origin of trophic novelties in coral reef fishes. Journal of Systematic Palaeontology, 16, 1189–1219. doi:10.1080/14772019.2017.1371257 30210265 10.1080/14772019.2017.1371257 PMC6130837

[CIT0084] Marramà G., Klug S., Vos De, J. & Kriwet J. 2018a Anatomy, relationships and palaeobiogeographic implications of the first Neogene holomorphic stingray (Myliobatiformes, Dasyatidae) from the early Miocene of Sulawesi, Indonesia, SE Asia. Zoological Journal of the Linnean Society, doi:10.1093/zoolinnean/zly020

[CIT0085] Marramà G., Engelbrecht A., Mörs T., Reguero M. A. & Kriwet J. 2018b The southernmost occurrence of *Brachycarcharias* (Lamniformes, Odontaspididae) from the Eocene of Antarctica provides new information about the paleobiogeography and paleobiology of Paleogene sand tiger sharks. Rivista Italiana di Paleontologia e Stratigrafia, 124(2), 283–298.

[CIT0086] Marramà G., Carnevale G., Engelbrecht A., Claeson K. M., Zorzin R., Fornasiero M. & Kriwet J. 2018c A synoptic review of the Eocene (Ypresian) cartilaginous fishes (Chondrichthyes: Holocephali, Elasmobranchii) of the Bolca Konservat-Lagerstätte, Italy. *Paläontologische Zeitschrift*, 29, 283–313. 10.1007/s12542-017-0387-z PMC5970259 29875508

[CIT0087] McEachran J. D. & Carvalho M. R. 2002 Rajidae. Pp. 531–561 in Carpenter K. E. (ed.) *The living marine resources of the Western Central Atlantic. Volume 1: Introduction, molluscs, crustaceans, hagfishes, sharks, batoid fishes, and chimaeras. FAO Species identification guide for fishery purposes and American Society of Ichthyologists and Herpetologists. Special Publication No. 5*. FAO, Rome.

[CIT0087a] McEachran J. D. & Compagno L. G. V. 1982 Interrelationships of and within *Breviraja* based on anatomical structures (Pisces: Rajoidei). Bulletin of Marine Science, 32(2), 399–425.

[CIT0088] McEachran J. D. & Dunn K. A. 1998 Phylogenetic analysis of skates, a morphologically conservative clade of elasmobranchs (Chondrichthyes: Rajidae). Copeia, 1998, 271–290.

[CIT0089] McEachran J. D. & Konstantinou H. 1996 Survey of the variation in alar and malar thorns in skates: phylogenetic implications (Chondrichthyes: Rajoidei). Journal of Morphology, 228, 165–178. 29852593 10.1002/(SICI)1097-4687(199605)228:2<165::AID-JMOR5>3.0.CO;2-4

[CIT0090] McEachran J. D. & Miyake T. 1990a Phylogenetic interrelationships of skates: a working hypothesis (Chondrichthyes: Rajoidei). Pp. 285–304 in Pratt H. L. Jr., Gruber S. H. & Taniuchi T. (eds) Elasmobranchs as living resources: advances in the biology, ecology, systematics, and the status of the fisheries. NOAA Tech Report NMFS 90.

[CIT0091] McEachran J. D. & Miyake T. 1990b Zoogeography and bathymetry of skates (Chondrichthyes, Rajoidei). Pp. 305–326 in Pratt H. L. Jr., Gruber S. H. & Taniuchi T. (eds) *Elasmobranchs as Living Resources: Advances in the Biology, Ecology, Systematics, and the Status of the Fisheries*. NOAA Tech Report NMFS 90.

[CIT0092] McEachran J. D., Dunn K. A. & Miyake T. 1996 Interrelationships of the batoid fishes (Chondrichthyes: Batoidea). Pp. 63–82 in Stiassny M. J., Parenti L. R. & Johnson G. D. (eds) Interrelationships of fishes. Academic Press, London.

[CIT0093] Miyake T., Vaglia J. L., Taylor L. H. & Hall B. K. 1999 Development of dermal denticles in skates (chondrichthyes, batoidea): patterning and cellular differentiation. Journal of Morphology, 241, 61–81. 10398324 10.1002/(SICI)1097-4687(199907)241:1<61::AID-JMOR4>3.0.CO;2-S

[CIT0094] Müller A. 1999 Ichthyofaunen aus dem atlantischen Tertiar der USA. *Leipziger Geowiss*, **9–10**, 1–360.

[CIT0095] Müller A. & Rozenberg A. 2003 Fischreste aus dem Unteroligozän der Krim. Neues *Jahrbuch für Geologie und Paläontologie, Monatshefte*, 2003, 321–339.

[CIT0096] Naylor G. J. P., Caira J. N., Jensen K., Rosana A. M., Straube N. &Lakner,. 2012a Elasmobranch phylogeny: a mitochondrial estimate based on 595 species. Pp. 31–56 in Carrier J. C., Musick J. A. & Heithaus M. R. (eds) Biology of sharks and their relatives. 2nd edition CRC Press, Boca Raton.

[CIT0097] Naylor G. J. P., Caira J. N., Jensen K., Rosana A. M., White W. T. & Last P. R. 2012b A DNA sequence-based approach to the identification of shark and rays species and its implication of global elasmobranch diversity and parasitology. Bulletin of the American Museum of Natural History, 367, 1–262.

[CIT0098] Nishida K. 1990 Phylogeny of the suborder Myliobatoidei. Hokkaido University Fisheries Memoir, 37, 1–108.

[CIT0099] Piller W. E., Harzhauser M. & Mandic O. 2007 Miocene Central Paratethys stratigraphy – current status and future directions. Stratigraphy, 4, 151–168.

[CIT0100] Pollerspöck J. & Straube N. 2017 A new deep-sea elasmobranch fauna from the Central Paratethys (Neuhofener Beds, Mitterdorf, near Passau, Germany, Early Miocene, Middle Burdigalian). Zitteliana, 90, 27–54.

[CIT0101] Prasad G. V. R. & Cappetta H. 1993 Late Cretaceous selachians from India and the age of the Deccan Traps. Palaeontology, 36, 231–248.

[CIT0102] Purdy R. W., Schneider V. P., Applegate S. P., McLellan J. H., Meyer R. L. & Slaughter B. H. 2001 The Neogene sharks, rays, and bony fishes from Lee Creek Mine, Aurora, North Carolina. Smithsonian Contributions to Paleobiology, 90, 71–202.

[CIT0103] Radwanski A. 1965 A contribution to the knowledge of Miocene Elasmobranchii from Pinczow (Poland). Acta Palaeontologica Polonica, 10, 267–276.

[CIT0104] Ray C. E., Wetmore A., Dunkle D. H. & Drez P. 1968 Fossil vertebrates from the marine Pleistocene of southeastern Virginia. Smithsonian Miscellaneous Collections, 153(3), 1–25.

[CIT0105] Reinecke T. 2015 Batoids (Rajiformes, Torpediniformes, Myliobatiformes) from the Sülstorf Beds (Chattian, Late Oligocene) of Mecklenburg, northeastern Germany: a revision and description of three new species. *Palaeovertebrata*, 39(2), e2.

[CIT0106] Reinecke T., Moths H., Grant A. & Breitkreutz H. 2005 Die Elasmobranchier des Norddeutschen Chattiums, insbesondere des Sternberger Gesteins (Eochattium, Oberes Oligozän). Palaeontos, 8, 1–134.

[CIT0107] Rögl F. 1998 Palaeogeographic considerations for Mediterranean and Paratethys seaways (Oligocene to Miocene). Annalen des Naturhistorischen Museums in Wien, 99(A), 279–310.

[CIT0108] Rögl F., Schultz O. & Hölz O. 1973 Beschreibung des Holostratotypus und der Faziostratitypen – A. Holostratotypus und Faziostratitypen der Innviertler Schichtengruppe. Pp. 140–196 in Papp A., Rögl F. & Senes J. (eds) *M2 Ottnangien. Die Innviertler, Salgótarjáner, Bántapusztaer Schichtengruppe und die Rzehakia Formation. Chronostratigraphie und Neostratotypen, Miozän der Zentralen Paratethys*. Verlag der Slowakischen Akademie der Wissenschaften, Bratislava.

[CIT0109] Roth F. & Hoedemakers K. 2005 The marine Gram Formation at Gram, Denmark: late Miocene geology and palaeontology. Palaeontos, 7, 1–189.

[CIT0110] Rupp C., Hofmann T., Jochum B., Pfleiderer S., Schedl A., Schindlbauer G., Schubert G., Slapansky P., Tilch N., van Husen D., Wagner L. & Wimmer-Frey I. 2008 Geologische Karte der Republik Österreich 1:50.000, Blatt 47 Ried im Innkreis. Erläuterungen zu Blatt 47 Riedim Innkreis. Geological Survey of Austria, Vienna.

[CIT0111] Sabol M. & Kovac M. 2006 Badenian palaeoenvironment, faunal succesion and biostratigraphy: a case study from Northern Vienna Basin, Devinska Nova Ves-Bonanza site (Western Carpathians, Slovakia). Beitraäge zur Palaäontologie, 30, 415–425.

[CIT0112] Sahni A. & Mehrotra D. K. 1981 The elasmobranch fauna of coastal Miocene sediments of peninsular India. *Biological Memoirs Lucknow*, **5**(2), 83–121.

[CIT0113] Schaefer J. T. & Summers A. P. 2005 Batoid wing skeletal structure: novel morphologies, mechanical implications, and phylogenetic patterns. Journal of Morphology, 264, 298–313. 15838841 10.1002/jmor.10331

[CIT0114] Schultz O. 1979 Supplementary notes on elasmobranch and teleost fish remains from the Korytnica Clays (Middle Miocene; Holy Cross Mountains, Central Poland). *Acta Geologica Polonica*, 29, 287–293.

[CIT0115] Schultz O. 2013 Pisces. Pp. 1– 576 in W. E. Piller (ed.) *Catalogus Fossilium Austriae, Band 3*. Verlag der Österreichischen Akademie der Wissenschaften, Wien.

[CIT0116] Schultz O., Brzobohatý R. & Kroupa O. 2010 Fish teeth from the Middle Miocene of Kienberg at Mikulov, Czech Republic, Vienna Basin. Annalen des Naturhistorischen Museums in Wien, 112, 489–506.

[CIT0117] Scotese C. R. 2002 *Paleomap*. Updated at: http://www.scotese.com, accessed 9 April 2018.

[CIT0118] Siverson M. & Cappetta H. 2001 A skate in the lowermost Maastrichtian of Southern Sweden. Palaeontology, 44, 431–445.

[CIT0120] Steininger F. V. 1966 Zur Kenntnis fossiler Euselachier-Eikapseln aus dem Ober-Oligozan von Mitteleuropa. *Mitteilungen der Bayerischen Staatssammlung für Paläontologie und histor. Geologie*, **6**, 37–49.

[CIT0121] Steurbaut E. & Herman J. 1978 Biostratigraphie et poissons fossils de la formation de l’Argile de Boom (Oligocène moyen du bassin belge). *Géobios*, 11, 297–325.

[CIT0122] Underwood C. J., Johanson Z., Welten M., Metscher B., Rasch L. J., Fraser G. J. & Smith M. M. 2015 Development and evolution of dentition pattern and tooth order in the skates and rays (Batoidea; Chondrichthyes). PLoS ONE, 10(4), e0122553. 25874547 10.1371/journal.pone.0122553 PMC4398376

[CIT0123] Valsecchi E., Pasolini P., Bertozzi M., Garoia F., Ungaro N., Vacchi M., Sabelli B. & Tinti F. 2005 Rapid Miocene–Pliocene dispersal and evolution of Mediterranean rajid fauna as inferred by mitochondrial gene variation. Journal of Evolutionary Biology, 18, 436–446. 15715849 10.1111/j.1420-9101.2004.00829.x

[CIT0124] Walker M. B. & Kimmel C. B. 2007 A two-color acid-free cartilage and bone stain for zebrafish larvae. Biotechnic & Histochemistry, 82(1), 23–28. 17510811 10.1080/10520290701333558

[CIT0501] Ward D. J. 1984 Additions to the fish fauna of the English Palaeogene. 5. A new species of *Raja* from the London Clay. *Tertiary Research*, 6(2), 65–68.

[CIT0125] Ward D. J. & Bonavia C. G. 2001. Additions to and review of the Miocene shark and ray fauna of Malta. *The Central Mediterranean Naturalist*, 3(3), 131–146.

[CIT0126] Welton B. J. 1972 Fossil sharks in Oregon. *The Ore Bin*, 34, 161–170.

[CIT0127] Welton B. J. & Farish R. F. 1993 The Collector’s Guide to Fossil Sharks and Rays from the Cretaceous of Texas. Before Time, Dallas, 204 pp.

[CIT0128] Werner C. 1989 Die Elasmobranchier-Fauna des Gebel Dist Member der Bahariya Formation (Obercenoman) der Oase Bahariya, Ägypten. Palaeo Ichthyologica, 5, 1–112.

[CIT0129] Wijnker E., Bor T. J., Wesselingh F. P., Munsterman D. K., Brinkhuis H., Burger A. W., Vonhof H. B., Post K., Hoedemakers K., Janse A. C. & Taverne N. 2008 Neogene stratigraphy of the Langenboom locality (Noord-Brabant, Netherlands). Netherlands Journal of Geosciences, 87(2), 165–180.

