# Supplementary material for: A new Miocene skate from the Central Paratethys (Upper Austria): the first unambiguous skeletal record for the Rajiformes (Chondrichthyes: Batomorphii)
Source: J Syst Palaeontol. 2018 Oct 30;17(11):937–60. doi: 10.1080/14772019.2018.1486336 (PMC6510527; doi:10.1080/14772019.2018.1486336)
Supplement: Supplemental_Appendix_B.docx [file TJSP_A_1486336_SM3276.docx]

**Appendix B.** Data matrix used in the phylogenetic analysis. Abbreviations: AAA, Amphi-American assemblage; NPA, North-Pacific assemblage.

| **Taxon** | **1** | **2** | **3** | **4** | **5** | **6** | **7** | **8** | **9** | **10** | **11** | **12** | **13** | **14** | **15** | **16** | **17** | **18** | **19** | **20** | **21** | **22** | **23** | **24** | **25** | **26** | **27** | **28** | **29** | **30** |
| --- | --- | --- | --- | --- | --- | --- | --- | --- | --- | --- | --- | --- | --- | --- | --- | --- | --- | --- | --- | --- | --- | --- | --- | --- | --- | --- | --- | --- | --- | --- |
| *Zapterix* | 0 | 0 | 0 | 0 | 0 | 0 | 0 | 0 | 0 | 0 | 0 | 0 | 0 | 0 | 0 | ? | ? | ? | ? | ? | ? | ? | 0 | ? | 0 | ? | 0 | 0 | 0 | 0 |
| *Rhinobatos* | 0 | 0 | 0 | 0 | 0 | 0 | 0 | 0 | 0 | 0 | 0 | 0 | 0 | 0 | 0 | ? | ? | ? | ? | ? | ? | ? | 0 | ? | 0 | ? | 0 | 0 | 0 | 0 |
| *Trygonorrhina* | 0 | 0 | 0 | 0 | 0 | ? | ? | 1 | 0 | 0 | 0 | 0 | 0 | 0 | 0 | ? | ? | ? | ? | ? | ? | ? | 0 | ? | 0 | ? | 0 | 0 | 0 | 0 |
| *Amblyraja* | 1 | 1 | 1 | 1 | 1 | 1 | 1 | 1 | 0 | 0 | [01] | [01] | 0 | 0 | 1 | 0 | 0 | 1 | 1 | 1 | 1 | 0 | 0 | ? | 1 | 1 | 1 | 0 | 1 | 0 |
| *Arhynchobatis* | 1 | 1 | 1 | 1 | 1 | ? | 1 | 1 | 1 | 0 | 0 | 0 | 2 | 0 | 1 | 1 | 1 | 1 | 1 | 1 | 1 | 1 | 1 | 0 | 1 | 0 | 4 | 1 | 1 | 0 |
| *Atlantoraja* | 1 | 1 | 1 | 1 | 1 | 1 | 1 | 1 | 0 | 0 | 1 | 0 | 0 | 0 | 1 | 1 | 1 | 0 | 1 | 1 | 1 | 1 | 0 | ? | 0 | ? | [02] | 0 | 1 | 0 |
| *Bathyraja* | 1 | 1 | 1 | 1 | 1 | 1 | 1 | 1 | 0 | 0 | 0 | 0 | 0 | 0 | 1 | 1 | 1 | 1 | 1 | 1 | 1 | 1 | 0 | ? | 1 | 0 | [34] | 1 | 1 | 0 |
| *Beringraja* NPA | 1 | 1 | 1 | 1 | 1 | 1 | 1 | 1 | 0 | 0 | 1 | 0 | 0 | 0 | 1 | 0 | 0 | 1 | 1 | 0 | 0 | 1 | 1 | 1 | 0 | ? | 0 | 0 | 0 | 0 |
| *Breviraja* | 1 | 1 | 1 | 1 | 1 | 1 | 1 | 1 | 0 | 0 | 0 | 1 | 1 | 0 | 1 | 0 | 0 | 1 | 1 | 1 | 1 | 0 | 1 | 1 | 1 | 1 | 1 | 0 | 0 | 0 |
| *Brochiraja* | 1 | 1 | 1 | 1 | 1 | 1 | 1 | 1 | 0 | 0 | 0 | 0 | 2 | 0 | 1 | 1 | 1 | 1 | 1 | 1 | 1 | 1 | 1 | 0 | 1 | 0 | 5 | 0 | 1 | 1 |
| *Cruriraja* | 1 | 1 | 1 | 1 | 1 | 1 | 1 | 1 | 0 | 1 | [01] | [01] | [01] | 0 | 1 | 0 | 1 | 1 | 1 | 0 | 1 | 1 | 1 | 1 | 0 | ? | 0 | 0 | 0 | 0 |
| *Dactylobatus* | 1 | 1 | 1 | 1 | 1 | 1 | 1 | 1 | 0 | 0 | 0 | 1 | 1 | 1 | ? | ? | ? | ? | ? | ? | ? | ? | ? | ? | 1 | 1 | 1 | 0 | 1 | 0 |
| *Dentiraja* | 1 | 1 | 1 | 1 | 1 | 1 | 1 | 1 | 0 | 0 | 1 | 0 | 0 | 0 | 1 | 0 | 0 | 1 | 1 | 0 | 0 | 1 | 1 | 1 | 0 | ? | 0 | 0 | 0 | 0 |
| *Dipturus* | 1 | 1 | 1 | 1 | 1 | 1 | 1 | 1 | 0 | 0 | 1 | 0 | 0 | 0 | 1 | 0 | 0 | 1 | 1 | 0 | 0 | 1 | 0 | ? | 0 | ? | 0 | 0 | 0 | 0 |
| *Fenestraja* | 1 | 1 | 1 | 1 | 1 | ? | 1 | 1 | 0 | 0 | 0 | 0 | 0 | 0 | 1 | 0 | 1 | 1 | 1 | 1 | 1 | 1 | 1 | 1 | 1 | 2 | 2 | 0 | 1 | 1 |
| *Gurgesiella* | 1 | 1 | 1 | 1 | 1 | ? | 1 | 1 | 0 | 0 | 0 | 0 | 2 | 0 | 1 | 0 | 1 | 1 | 1 | 1 | 1 | 1 | 1 | 1 | 1 | 2 | 2 | 0 | 1 | 1 |
| *Hongeo* | 1 | 1 | 1 | 1 | 1 | 1 | 1 | 1 | 0 | 0 | 1 | 0 | 0 | 0 | 1 | 0 | 0 | 1 | 1 | 0 | 0 | ? | 1 | 1 | 0 | 0 | 1 | 2 | 0 | 0 |
| *Irolita* | 1 | 1 | 1 | 1 | 1 | ? | 1 | 1 | 1 | 0 | 1 | 0 | 0 | 0 | 1 | 1 | 1 | 1 | 1 | 1 | 1 | 1 | 0 | ? | 1 | 2 | 4 | 1 | 1 | 1 |
| *Leucoraja* | 1 | 1 | 1 | 1 | 1 | 1 | 1 | 1 | 0 | 0 | [01] | [01] | [01] | [01] | 1 | 0 | 0 | 1 | 1 | 1 | 1 | 0 | 1 | 1 | [01] | ? | [01] | 0 | 1 | 0 |
| *Malacoraja* | 1 | 1 | 1 | 1 | 1 | 1 | 1 | 1 | 0 | 0 | 0 | 0 | 0 | 0 | 1 | 0 | 0 | 1 | 1 | 1 | 1 | 1 | 1 | 1 | 1 | 0 | 0 | 0 | 1 | 0 |
| *Neoraja* | 1 | 1 | 1 | 1 | 1 | 1 | 1 | 1 | 0 | 0 | 0 | 0 | 0 | 0 | 1 | 0 | 0 | 1 | 1 | 1 | 1 | 1 | 1 | 1 | 1 | 2 | 2 | 0 | 1 | 0 |
| *Notoraja* | 1 | 1 | 1 | 1 | 1 | 1 | 1 | 1 | 0 | 0 | 0 | 0 | 2 | 0 | 1 | 1 | 1 | 1 | 1 | 1 | 1 | 1 | 1 | 0 | 1 | 0 | 5 | 0 | 1 | 1 |
| *Okamejei* | 1 | 1 | 1 | 1 | 1 | 1 | 1 | 1 | 0 | 0 | 1 | 0 | 0 | 0 | 1 | 0 | 0 | 1 | 1 | 0 | 0 | 1 | 1 | 1 | 0 | ? | 0 | 0 | 0 | 0 |
| *Ostarriraja* | ? | ? | ? | ? | ? | ? | ? | ? | ? | 0 | ? | ? | ? | ? | ? | ? | ? | ? | ? | ? | ? | ? | ? | ? | ? | ? | ? | ? | 1 | 0 |
| *Pavoraja* | 1 | 1 | 1 | 1 | 1 | 1 | 1 | 1 | 0 | 0 | 0 | 0 | 2 | 0 | 1 | 1 | 1 | 1 | 1 | 1 | 1 | 1 | 1 | 0 | 1 | 0 | 5 | 0 | 1 | 1 |
| *Psammobatis* | 1 | 1 | 1 | 1 | 1 | ? | 1 | 1 | 1 | 0 | 0 | [01] | [01] | 0 | 1 | 1 | 1 | 1 | 0 | 1 | 1 | 1 | [01] | 0 | 1 | 0 | 5 | 1 | 1 | 1 |
| *Pseudoraja* | 1 | 1 | 1 | 1 | 1 | ? | 1 | 1 | 1 | 0 | 0 | 0 | 0 | 0 | ? | ? | ? | ? | ? | ? | ? | ? | ? | ? | 1 | 0 | 5 | 0 | 1 | 1 |
| *Raja* | 1 | 1 | 1 | 1 | 1 | 1 | 1 | 1 | 0 | 0 | 1 | 0 | 0 | 0 | 1 | 0 | 0 | 1 | 1 | 0 | 0 | 1 | 1 | 1 | 0 | ? | 0 | 0 | 0 | 0 |
| *Rajella* | 1 | 1 | 1 | 1 | 1 | 1 | 1 | 1 | 0 | 0 | 0 | 1 | 1 | 0 | 1 | 0 | 0 | 1 | 1 | 1 | 1 | 0 | 1 | 1 | 1 | 1 | 1 | 0 | 0 | 0 |
| *Rhinoraja* | 1 | 1 | 1 | 1 | 1 | 1 | 1 | 1 | 0 | 0 | 0 | 0 | 0 | 0 | 1 | 1 | 1 | 1 | 1 | 1 | 1 | 1 | 0 | ? | 1 | 0 | 4 | 1 | 1 | 0 |
| *Rioraja* | 1 | 1 | 1 | 1 | 1 | 1 | 1 | 1 | 0 | 0 | 1 | 0 | 0 | 0 | 1 | 1 | 1 | 0 | 1 | 1 | 1 | 1 | 0 | ? | 0 | ? | 0 | 0 | 1 | 0 |
| *Rostoraja* | 1 | 1 | 1 | 1 | 1 | 1 | 1 | 1 | 0 | 0 | 1 | 0 | 0 | 0 | ? | ? | ? | ? | ? | ? | ? | ? | ? | ? | 0 | ? | 0 | 0 | 0 | 0 |
| *‘Rostroraja'* AAA | 1 | 1 | 1 | 1 | 1 | 1 | 1 | 1 | 0 | 0 | 1 | 0 | 0 | 0 | 1 | 0 | 0 | 1 | 1 | 0 | 0 | 1 | 1 | 1 | 0 | ? | 0 | 0 | 0 | 0 |
| *Schroederobatis* | 1 | 1 | 1 | 1 | 1 | 1 | 1 | 1 | 2 | 1 | 1 | 0 | 0 | 0 | 1 | 0 | 1 | 0 | 1 | ? | ? | ? | 0 | ? | 0 | ? | 0 | 0 | 0 | 0 |
| *Springeria* | 1 | 1 | 1 | 1 | 1 | 1 | 1 | 1 | 2 | 1 | 1 | 0 | 0 | 0 | 1 | 0 | 1 | 0 | 1 | 0 | 1 | 1 | 0 | ? | 0 | ? | 1 | 0 | 0 | 0 |
| *Sympterygia* | 1 | 1 | 1 | 1 | 1 | ? | 1 | 1 | 0 | 0 | 0 | 0 | 0 | 0 | 1 | 1 | 1 | 1 | 0 | 1 | 1 | 1 | 0 | ? | 1 | 0 | 4 | 1 | 1 | 0 |
|  |  |  |  |  |  |  |  |  |  |  |  |  |  |  |  |  |  |  |  |  |  |  |  |  |  |  |  |  |  |  |
|  |  |  |  |  |  |  |  |  |  |  |  |  |  |  |  |  |  |  |  |  |  |  |  |  |  |  |  |  |  |  |
| **Taxon** | **31** | **32** | **33** | **34** | **35** | **36** | **37** | **38** | **39** | **40** | **41** | **42** | **43** | **44** | **45** | **46** | **47** | **48** | **49** | **50** | **51** | **52** | **53** | **54** | **55** | **56** | **57** | **58** | **59** | **60** |
| *Zapterix* | 0 | 0 | 0 | 0 | 0 | 0 | 0 | 0 | 0 | 0 | 0 | 0 | 0 | 0 | 0 | 0 | 0 | 0 | 0 | 0 | 0 | 0 | 0 | 0 | 0 | 0 | 0 | 0 | 0 | ? |
| *Rhinobatos* | 0 | 0 | 0 | 0 | 0 | 0 | 0 | 0 | 0 | 0 | 0 | 0 | 0 | 0 | 0 | 0 | 0 | 0 | 0 | 0 | 0 | 0 | 0 | 0 | 0 | 0 | 0 | 0 | 0 | 1 |
| *Trygonorrhina* | 0 | 0 | 0 | 0 | 0 | 0 | 0 | 0 | 0 | ? | ? | ? | ? | ? | ? | 0 | 0 | ? | ? | ? | ? | 0 | 0 | 0 | ? | ? | 0 | 0 | 0 | ? |
| *Amblyraja* | 0 | 0 | 0 | 2 | 1 | 0 | 0 | 0 | 0 | 1 | 1 | 0 | 0 | 0 | 0 | 1 | 2 | 0 | 0 | 0 | 1 | 3 | 2 | 2 | 0 | 0 | 1 | 0 | 0 | 0 |
| *Arhynchobatis* | 0 | 0 | 1 | 0 | 0 | 1 | 0 | 1 | 0 | 0 | 0 | 1 | 0 | 0 | 1 | 1 | 2 | 0 | 1 | ? | 2 | 2 | 1 | 2 | 0 | 1 | 0 | 1 | 0 | 0 |
| *Atlantoraja* | 0 | 1 | 1 | 1 | 0 | 1 | 0 | 0 | 0 | 0 | 0 | 0 | 0 | 0 | 1 | 2 | ? | 0 | 0 | 0 | 0 | 1 | 1 | 2 | 0 | 0 | 0 | 3 | 1 | 1 |
| *Bathyraja* | 0 | 0 | 1 | 0 | 0 | 1 | 0 | 1 | 0 | 0 | 0 | 0 | 0 | 0 | 1 | 3 | 2 | 0 | 0 | 1 | 0 | 2 | 1 | 2 | 0 | 0 | 0 | [01] | 0 | 0 |
| *Beringraja* NPA | 0 | 0 | 0 | 2 | 0 | 1 | 1 | 0 | 0 | 1 | 1 | 0 | 0 | 0 | 0 | 1 | 1 | 0 | 0 | 0 | 0 | 3 | 1 | 1 | 1 | 0 | 1 | 4 | ? | ? |
| *Breviraja* | 0 | 2 | 0 | 2 | 1 | 0 | 0 | 1 | 0 | 1 | 1 | 0 | 1 | 1 | 0 | 1 | 2 | 1 | 0 | 0 | 1 | 3 | 2 | 2 | 0 | 0 | 1 | 0 | 1 | 1 |
| *Brochiraja* | 1 | 0 | 1 | 0 | 0 | 1 | 0 | 1 | 0 | 0 | 0 | 1 | 0 | 0 | 1 | 3 | 2 | 0 | 0 | 0 | 0 | 2 | 1 | 2 | 0 | 1 | ? | ? | ? | 1 |
| *Cruriraja* | 0 | 2 | 0 | 2 | 0 | 1 | 0 | 0 | 0 | 1 | 1 | 0 | 0 | 0 | 0 | 1 | 1 | 0 | 0 | 0 | 0 | 3 | 1 | 2 | 0 | 0 | 1 | 3 | 0 | 1 |
| *Dactylobatus* | 0 | 0 | 0 | 2 | 0 | 1 | 0 | 0 | 0 | 1 | 1 | 0 | 1 | 1 | 0 | 1 | 2 | 0 | 0 | 0 | 1 | 3 | 0 | 2 | 0 | 0 | 1 | 0 | 0 | 0 |
| *Dentiraja* | 0 | 0 | 0 | 2 | 0 | 1 | 1 | 0 | 0 | 1 | 1 | 0 | 0 | 0 | 0 | 1 | 1 | 0 | 0 | 0 | 0 | 3 | 1 | 1 | 1 | 0 | 1 | 4 | ? | ? |
| *Dipturus* | 0 | 0 | 0 | 2 | 0 | 1 | 1 | 0 | 0 | 1 | 1 | 0 | 0 | 0 | 0 | 1 | 1 | 0 | 0 | 0 | 0 | 3 | 1 | 1 | [01] | 0 | 1 | 4 | 0 | 0 |
| *Fenestraja* | 2 | 2 | 0 | 2 | 0 | 1 | 0 | 1 | 0 | 1 | 1 | 0 | 0 | 0 | 0 | 1 | 2 | 0 | 1 | 0 | 0 | 3 | 1 | 2 | 0 | 0 | 1 | 1 | 1 | 1 |
| *Gurgesiella* | 2 | 2 | 0 | 2 | 0 | 1 | 0 | 1 | 0 | 1 | 1 | 0 | 0 | 0 | 0 | 1 | 2 | 0 | 1 | 0 | 0 | 3 | 1 | 2 | 0 | 0 | 1 | 1 | 1 | 1 |
| *Hongeo* | 0 | 0 | 0 | 2 | 1 | 1 | 1 | 0 | 0 | 1 | 1 | 0 | 0 | 0 | 0 | 1 | 2 | 0 | 0 | 0 | 0 | 3 | 1 | 1 | 0 | 0 | ? | ? | ? | ? |
| *Irolita* | 0 | 0 | 1 | 1 | 0 | 1 | 0 | 1 | 0 | 0 | 0 | 1 | 0 | 0 | 1 | 3 | 2 | 0 | 1 | ? | 2 | 2 | 1 | 2 | 0 | 1 | ? | ? | 1 | 1 |
| *Leucoraja* | 0 | 0 | 0 | 2 | 0 | 1 | [01] | 0 | 0 | 1 | [01] | 0 | [01] | [01] | 0 | 1 | [12] | 0 | 0 | 0 | [12] | 3 | [12] | [12] | [01] | 0 | 1 | 1 | 0 | 0 |
| *Malacoraja* | 1 | 2 | 0 | 2 | 1 | 1 | 0 | 0 | 0 | 1 | 1 | 0 | 0 | 0 | 0 | 1 | 2 | 0 | 0 | 0 | 0 | 3 | 1 | 2 | 0 | 0 | 1 | 0 | 1 | 1 |
| *Neoraja* | 1 | 2 | 0 | 2 | 1 | 1 | 0 | 1 | 0 | 1 | 1 | 0 | 0 | 0 | 0 | 1 | 2 | 0 | 0 | 0 | 0 | 3 | 1 | 2 | 0 | 0 | ? | ? | 0 | 1 |
| *Notoraja* | 1 | 0 | 1 | 0 | 0 | 1 | 0 | 1 | 0 | 0 | 0 | 1 | 0 | 0 | 1 | 3 | 2 | 0 | 0 | 0 | 0 | 2 | 1 | 2 | 0 | 1 | 0 | 2 | 0 | 0 |
| *Okamejei* | 0 | 0 | 0 | 2 | 0 | 1 | 1 | 0 | 0 | 1 | 1 | 0 | 0 | 0 | 0 | 1 | 1 | 0 | 0 | 0 | 0 | 3 | 1 | 1 | 1 | 0 | ? | ? | 0 | 0 |
| *Ostarriraja* | 0 | ? | ? | ? | 0 | ? | ? | ? | 0 | ? | ? | ? | ? | ? | ? | ? | ? | ? | ? | ? | ? | ? | ? | ? | ? | ? | ? | ? | 0 | ? |
| *Pavoraja* | 1 | 0 | 1 | 0 | 0 | 1 | 0 | 1 | 0 | 0 | 0 | 1 | 0 | 0 | 1 | 3 | 2 | 0 | 0 | 0 | 0 | 2 | 1 | 2 | 0 | 1 | 0 | 2 | 0 | 1 |
| *Psammobatis* | 0 | 0 | 1 | 0 | [01] | 0 | 0 | 1 | 1 | 0 | 0 | 0 | 0 | 0 | [01] | 2 | ? | 0 | [01] | ? | 2 | 1 | 1 | 2 | 0 | 0 | 0 | 1 | 1 | 1 |
| *Pseudoraja* | 1 | 0 | 1 | 1 | 0 | 0 | 0 | 1 | 0 | ? | ? | ? | ? | ? | ? | ? | ? | ? | ? | ? | ? | ? | 0 | ? | ? | ? | 0 | 2 | 0 | 0 |
| *Raja* | 0 | 0 | 0 | 2 | 0 | 1 | 1 | 0 | 0 | 1 | 0 | 0 | 0 | 0 | 0 | 1 | 1 | 0 | 0 | ? | 2 | 3 | 1 | 1 | 1 | 0 | 1 | 4 | 0 | 1 |
| *Rajella* | 0 | 2 | 0 | 2 | [01] | 0 | 0 | 0 | 0 | 1 | 1 | 0 | 0 | 0 | 0 | 1 | 2 | 1 | 0 | 0 | 1 | 3 | 2 | 2 | 0 | 0 | 1 | 0 | 0 | 1 |
| *Rhinoraja* | 0 | 0 | 1 | 0 | 0 | 1 | 0 | 1 | 0 | 0 | 0 | 0 | 0 | 0 | 1 | 3 | 2 | 0 | 0 | 1 | 0 | 2 | 1 | 2 | 0 | 0 | 0 | 1 | 1 | 1 |
| *Rioraja* | 0 | 1 | 1 | 1 | 0 | 1 | 0 | 0 | 0 | 0 | 0 | 0 | 0 | 0 | 1 | 2 | ? | 0 | 0 | ? | 2 | 1 | 1 | 2 | 0 | 0 | 0 | 3 | 1 | 1 |
| *Rostoraja* | 0 | 0 | 0 | 2 | 0 | 1 | 1 | 0 | 0 | 1 | 0 | 0 | 0 | 0 | 0 | 1 | 1 | 0 | 0 | 0 | 1 | 3 | 1 | 1 | 1 | 0 | 1 | 4 | 0 | 0 |
| *‘Rostroraja'* AAA | 0 | 0 | 0 | 2 | 0 | 1 | 1 | 0 | 0 | 1 | 1 | 0 | 0 | 0 | 0 | 1 | 1 | 0 | 0 | 0 | 0 | 3 | 1 | 1 | 1 | 0 | 1 | 4 | ? | ? |
| *Schroederobatis* | 0 | 0 | 0 | 2 | 0 | 1 | 0 | 0 | 2 | 1 | 1 | 0 | 0 | 0 | 0 | 3 | ? | 0 | 0 | 0 | 0 | 3 | 1 | 2 | 0 | 0 | 1 | 3 | 1 | 1 |
| *Springeria* | 0 | 0 | 0 | 2 | 0 | 1 | 0 | 0 | 2 | 1 | 1 | 0 | 0 | 0 | 0 | 1 | 1 | 0 | 0 | 0 | 0 | 3 | 1 | 2 | 0 | 0 | 1 | 3 | 0 | 0 |
| *Sympterygia* | 0 | 0 | 1 | 0 | 0 | 1 | 0 | 1 | 1 | 0 | 0 | 0 | 0 | 0 | 1 | 3 | ? | 0 | 1 | ? | 2 | 1 | 1 | 2 | 0 | 0 | 0 | 1 | 1 | 1 |
|  |  |  |  |  |  |  |  |  |  |  |  |  |  |  |  |  |  |  |  |  |  |  |  |  |  |  |  |  |  |  |
|  |  |  |  |  |  |  |  |  |  |  |  |  |  |  |  |  |  |  |  |  |  |  |  |  |  |  |  |  |  |  |
| **Taxon** | **61** | **62** | **63** | **64** | **65** | **66** | **67** | **68** | **69** | **70** | **71** | **72** | **73** | **74** |  |  |  |  |  |  |  |  |  |  |  |  |  |  |  |  |
| *Zapterix* | ? | 0 | 0 | 0 | 0 | 0 | 0 | 0 | 1 | 0 | 0 | 0 | 0 | 0 |  |  |  |  |  |  |  |  |  |  |  |  |  |  |  |  |
| *Rhinobatos* | ? | 0 | 0 | 0 | 0 | 0 | 0 | 0 | 0 | 0 | 0 | 0 | 0 | 0 |  |  |  |  |  |  |  |  |  |  |  |  |  |  |  |  |
| *Trygonorrhina* | ? | 0 | 0 | 0 | 0 | 0 | 0 | 0 | 1 | 0 | 0 | 0 | 0 | 0 |  |  |  |  |  |  |  |  |  |  |  |  |  |  |  |  |
| *Amblyraja* | 1 | 1 | 1 | 0 | 0 | 0 | 1 | 0 | 0 | 0 | 1 | 1 | 1 | 2 |  |  |  |  |  |  |  |  |  |  |  |  |  |  |  |  |
| *Arhynchobatis* | 0 | 0 | 1 | 0 | 1 | 0 | 0 | 0 | 0 | 1 | 1 | 1 | 1 | ? |  |  |  |  |  |  |  |  |  |  |  |  |  |  |  |  |
| *Atlantoraja* | ? | 0 | 1 | 0 | 1 | 0 | 0 | 0 | 0 | 1 | 1 | 1 | 1 | 2 |  |  |  |  |  |  |  |  |  |  |  |  |  |  |  |  |
| *Bathyraja* | 1 | 0 | 1 | 0 | 0 | 0 | 1 | 0 | 0 | 1 | 1 | 1 | 1 | 2 |  |  |  |  |  |  |  |  |  |  |  |  |  |  |  |  |
| *Beringraja* NPA | ? | ? | ? | ? | ? | ? | ? | ? | ? | ? | 1 | 1 | 1 | 2 |  |  |  |  |  |  |  |  |  |  |  |  |  |  |  |  |
| *Breviraja* | ? | 0 | 1 | 0 | 0 | 0 | 0 | 0 | 0 | 1 | 1 | 1 | 1 | 2 |  |  |  |  |  |  |  |  |  |  |  |  |  |  |  |  |
| *Brochiraja* | ? | ? | 1 | 0 | 0 | 0 | 1 | 0 | 1 | ? | 1 | 1 | 1 | 2 |  |  |  |  |  |  |  |  |  |  |  |  |  |  |  |  |
| *Cruriraja* | 1 | 0 | 1 | 0 | 1 | 0 | 1 | 0 | 0 | 0 | 1 | 1 | 1 | ? |  |  |  |  |  |  |  |  |  |  |  |  |  |  |  |  |
| *Dactylobatus* | 1 | 0 | 1 | 0 | 0 | 0 | 1 | 0 | 1 | 0 | 1 | 1 | 1 | 2 |  |  |  |  |  |  |  |  |  |  |  |  |  |  |  |  |
| *Dentiraja* | ? | ? | ? | ? | ? | ? | ? | ? | ? | ? | 1 | 1 | 1 | ? |  |  |  |  |  |  |  |  |  |  |  |  |  |  |  |  |
| *Dipturus* | 0 | 0 | 1 | 0 | ? | 1 | 0 | 0 | 0 | 0 | 1 | 1 | 1 | 2 |  |  |  |  |  |  |  |  |  |  |  |  |  |  |  |  |
| *Fenestraja* | ? | 0 | 1 | 0 | 1 | 0 | 1 | 1 | 0 | 0 | 1 | 1 | 1 | ? |  |  |  |  |  |  |  |  |  |  |  |  |  |  |  |  |
| *Gurgesiella* | ? | 1 | 1 | 0 | 0 | 0 | 1 | 1 | 1 | 0 | 1 | 1 | 1 | 1 |  |  |  |  |  |  |  |  |  |  |  |  |  |  |  |  |
| *Hongeo* | ? | ? | ? | ? | ? | ? | ? | ? | ? | ? | 1 | 1 | 1 | 1 |  |  |  |  |  |  |  |  |  |  |  |  |  |  |  |  |
| *Irolita* | ? | 0 | 1 | 0 | 1 | 0 | 1 | 0 | 0 | 1 | 1 | 1 | 1 | 2 |  |  |  |  |  |  |  |  |  |  |  |  |  |  |  |  |
| *Leucoraja* | 1 | 0 | 1 | 0 | [01] | 1 | 1 | 0 | 1 | 0 | 1 | 1 | 1 | ? |  |  |  |  |  |  |  |  |  |  |  |  |  |  |  |  |
| *Malacoraja* | ? | 0 | 1 | 0 | 0 | 0 | 1 | 0 | 1 | 1 | 1 | 1 | 1 | 2 |  |  |  |  |  |  |  |  |  |  |  |  |  |  |  |  |
| *Neoraja* | ? | 0 | 1 | 0 | 0 | 0 | 1 | 0 | 0 | 0 | 1 | 1 | 1 | ? |  |  |  |  |  |  |  |  |  |  |  |  |  |  |  |  |
| *Notoraja* | 0 | 0 | 1 | 0 | 1 | 0 | 1 | 0 | 1 | 0 | 1 | 1 | 1 | ? |  |  |  |  |  |  |  |  |  |  |  |  |  |  |  |  |
| *Okamejei* | 0 | 0 | 1 | 0 | 1 | 0 | 1 | 0 | 0 | 0 | 1 | 1 | 1 | 2 |  |  |  |  |  |  |  |  |  |  |  |  |  |  |  |  |
| *Ostarriraja* | ? | 0 | 0 | ? | 1 | 0 | 1 | 0 | 1 | 0 | 1 | 1 | 1 | 0 |  |  |  |  |  |  |  |  |  |  |  |  |  |  |  |  |
| *Pavoraja* | ? | 0 | 1 | 0 | 1 | 0 | 1 | 0 | 1 | 0 | 1 | 1 | 1 | ? |  |  |  |  |  |  |  |  |  |  |  |  |  |  |  |  |
| *Psammobatis* | ? | 0 | 1 | 0 | 1 | 0 | 0 | 0 | 0 | 0 | 1 | 1 | 1 | 2 |  |  |  |  |  |  |  |  |  |  |  |  |  |  |  |  |
| *Pseudoraja* | 0 | 0 | 1 | 0 | 1 | 0 | 0 | 0 | 0 | 0 | 1 | 1 | 1 | ? |  |  |  |  |  |  |  |  |  |  |  |  |  |  |  |  |
| *Raja* | ? | 0 | 1 | 0 | 1 | 0 | 0 | 0 | 0 | 0 | 1 | 1 | 1 | 2 |  |  |  |  |  |  |  |  |  |  |  |  |  |  |  |  |
| *Rajella* | ? | 0 | 1 | 0 | 0 | 0 | 0 | 1 | 1 | 0 | 1 | 1 | 1 | 2 |  |  |  |  |  |  |  |  |  |  |  |  |  |  |  |  |
| *Rhinoraja* | 0 | 0 | 0 | 1 | 0 | 0 | 1 | 0 | 0 | 1 | 1 | 1 | 1 | 2 |  |  |  |  |  |  |  |  |  |  |  |  |  |  |  |  |
| *Rioraja* | ? | 1 | 1 | 0 | 1 | 0 | [01] | 0 | 0 | 0 | 1 | 1 | 1 | ? |  |  |  |  |  |  |  |  |  |  |  |  |  |  |  |  |
| *Rostoraja* | 0 | 0 | 0 | 1 | 1 | 1 | 0 | 1 | 1 | 0 | 1 | 1 | 1 | ? |  |  |  |  |  |  |  |  |  |  |  |  |  |  |  |  |
| *‘Rostroraja'* AAA | ? | ? | ? | ? | ? | ? | ? | ? | ? | ? | 1 | 1 | 1 | ? |  |  |  |  |  |  |  |  |  |  |  |  |  |  |  |  |
| *Schroederobatis* | ? | 1 | 1 | 0 | 1 | 0 | 0 | 1 | 0 | 1 | 1 | 1 | 1 | 2 |  |  |  |  |  |  |  |  |  |  |  |  |  |  |  |  |
| *Springeria* | 0 | 0 | 1 | 0 | 0 | 0 | 0 | 0 | 0 | 0 | 1 | 1 | 1 | 2 |  |  |  |  |  |  |  |  |  |  |  |  |  |  |  |  |
| *Sympterygia* | ? | 1 | 1 | 0 | 1 | 0 | 0 | 0 | 0 | 1 | 1 | 1 | 1 | 1 |  |  |  |  |  |  |  |  |  |  |  |  |  |  |  |  |
