# Supplementary material for: A new Miocene skate from the Central Paratethys (Upper Austria): the first unambiguous skeletal record for the Rajiformes (Chondrichthyes: Batomorphii)
Source: J Syst Palaeontol. 2018 Oct 30;17(11):937–60. doi: 10.1080/14772019.2018.1486336 (PMC6510527; doi:10.1080/14772019.2018.1486336)
Supplement: Supplemental_Appendix_A.docx [file TJSP_A_1486336_SM3275.docx]

**Appendix A.** List of morphological characters used for the phylogenetic analysis. Characters 1 to 58 are from McEachran & Dunn (1998) (with some modification; see **Remarks** below). We also included new dental characters (from 59 to 70) taken from Herman *et al*. (1994, 1995, 1996). Characters 71 to 73 are from Aschliman *et al*. (2012a), and character 74 is from Jeong & Nakabo (2009).

1. Development: (0) ovoviviparous; (1) oviparous.

2. Alar and/or malar thorns: (0) absent in mature males; (1) present in mature males.

3. Electric organs in lateral tail musculature: (0) absent; (1) present.

4. Second hypobranchial cartilage: (0) articulating with basibranchial copula; (1) fused with basibranchial copula.

5. Anterior portion of second hypobranchial cartilage: (0) present and articulating with second ceratobranchial cartilage; (1) absent and proximal section of second hypobranchial cartilage not articulating with second ceratobranchial cartilage.

6. Clasper skeleton with dorsal terminal cartilage: (0) on ventral aspect of clasper; (1) on dorsal aspect of clasper.

7. Clasper skeleton: (0) without ventral terminal cartilage; (1) with ventral terminal cartilage.

8. Anterior nasal lobe: (0) poorly developed and not completely covering naris; (1) expanded and connected to its antimere across a broad to narrow internarial space.

9. Tip of snout: (0) without terminal process; (1) with fleshy process, (2) with hairlike filament.

10. Pelvic fins: (0) anterior and posterior lobes continuous; (1) anterior and posterior lobes separated.

11. Squamation: (0) dorsal surface largely covered with dermal denticles; (1) dorsal surface largely naked.

12. Thorns over nuchal and scapular regions in distinct triangular patches: (0) absent; (1) present.

13. Thorns along dorsal midline of disc and tail: (0) in a single series; (1) in several series, with thorns of lateral series as large as those of medial series, (2) no midrow of thorns on disc and multiple rows of small thorns on tail. **Remarks**: this character, initially unordered in McEachran & Dunn (1998), is herein coded and analysed as ordered, in order to avoid loss of grouping information.

14. Midrow thorns along midline of disc and tail: (0) present in adults; (1) absent or largely absent in adults.

15. Alar thorns: (0) absent; (1) present. **Remarks**: Contrary to McEachran & Dunn (1998), who gave extra weight to the absence of the alar thorns by repeating the character several times (from their chs 15 to 21), we coded the absence/presence of alar thorns only once, and coded the state as unappicable (-) for the subsequent characters (from our chs 16 to 22) for those taxa which have no alar thorns. The presence of alar thorns in adult male skates (1) represents the derived condition. From ch. 16 to 22 the state (0) does not necessarly imply the basal condition in skates. We coded all these characters as unknown (?) for *Ostarriraja* gen. nov.

16. Alar thorns: (0) crown parallel to long axis of base; (1) crown oblique to long axis of base.

17. Alar thorns: (0) embedded; (1) exposed.

18. Alar thorns: (0) strongly reclined; (1) erect to slightly reclined.

19. Alar thorns: (0) neck partially enveloped with integument; (1) neck naked.

20. Crown of alar thorns: (0) with barb; (1) without barb.

21. Crown of alar thorns: (0) undulating; (1) linear.

22. Crown of alar thorns: (0) with keels running most of length; (1) with short or no keels.

23. Malar thorns: (0) absent; (1) present. **Remarks**: Contrary to McEachran & Dunn (1998), we separated the absence/presence of malar thorns and their origin in two different characters (23 and 24). The presence of malar thorns (1) is the derived condition of skates within batoids. However, malar thorns are secondarly lost in some skate genera (*Atlantoraja*, *Bathyraja*, *Irolita*, some *Psammobatis* species, *Rhinoraja*, *Rioraja*, *Sympterygia*, *Amblyraja*, *Schroederobatis*, *Springeria*, *Dipturus* (McEachran & Dunn 1998).

24. Malar thorns: (0) derived from denticles; (1) derived from enlarged placoid scales. **Remarks**: in this character, state (0) does not necessarly imply a basal condition in skates.

25. Rostral appendices: (0) attached over all or most of length to rostral cartilage; (1) largely free of rostral cartilage. **Remarks**: Contrary to McEachran & Dunn (1998, ch. 23), we separated the kind of attachment and shape of the rostral appendices in two different characters (chs 25 and 26), since the separation between the rostral appendices could be homologus.

26. Shape of rostral appendices: (0) narrow and depressed in cross section; (1) broad and triangular shaped, and depressed in cross section; (2) narrow and oval in cross-section. **Remarks**: in this character, a narrow and depressed rostral cartilage in cross section (0) does not necessarly imply the basal condition in skates.

27. Rostral cartilage: (0) continuous with neurocranium and stout over length; (1) continuous with neurocranium and stout proximally, but very slender and uncalcified distally (2) continuous with neurocranium and moderately stout proximally and attenuated distally (3) very slender and uncalcified over length; (4) very slender and segmented at base; (5) very slender and proximally attenuated.

28. Precerebral fontanelle: (0) narrow to moderately narrow and extending onto rostral cartilage distinctly anterior to leading edge of nasal capsules; (1) broad to extremely broad and not extending onto rostral cartilage appreciably anterior to leading edge of nasal capsules; (2) broad, extending forward onto the basal part of rostral cartilage slightly beyond leading edge of nasal capsule.**Remarks**: state (2) is taken from Jeong & Nakabo (2009) and is autapomorphic only for *Hongeo*.

29. Nasal capsules: (0) narrow and rectilinear; (1) broad and oval.

30. Nasal capsules: (0) without basal fenestrae; (1) with basal fenestrae.

31. Internasal plate: (0) broad; (1) narrow; (2) partially overlapping medial aspect of nasal capsules.

32. Preorbital processes: (0) moderately to well developed; (1) continuous with supraorbital crest; (2) poorly developed to degenerate and not continuous with supraorbital crest.

33. Basihyal: (0) without lateral projections; (1) with lateral projections.

34. Scapulocoracoid: (0) with stout anterior bridge; (1) with slender anterior bridge; (2) without anterior bridge.

35. Scapulocoracoid: (0) distance between pro- and mesocondyles less than distance between meso- and metacondyles; (1) distance between pro- and mesocondyles, and between meso- and metacondyles about equal. **Remarks:** Scapulocoracoid has been described and figured as elongated between the mesocondyle and the metacondyle (state 0) in guitarfishes *Rhinobatos*, *Zapteryx*, *Trygonorrhina*, and some skates by Nishida (1990, fig. 32), and McEachran *et al*. (1996, fig. 9), and this character was used in the phylogeny of Aschliman *et al*. (2012a). On the contrary, McEachran & Dunn (1998) reported for outgroups the condition of equidistance between scapular condyles. We follow in this case the scoring of Aschliman *et al.* (2012a), in which the basal condition (0, distance between pro- and mesocondyles less than distance between meso- and metacondyles) is imputed for outgroups and most of the skates, including *Ostarriraja* gen. nov*.* The derived condition (1, equidistance between condyles) is only present in *Amblyraja*, *Breviraja*, *Malacoraja*, *Neoraja*, and some species of *Rajella* and *Psammobatis* (McEachran & Dunn 1998).

36. Scapular process: (0) higher than dorsal margin of scapula; (1) little if any higher than dorsal margin of scapula.

37. Scapulocoracoid: (0) with postventral foramina; (1) with postventral fenestra.

38. Propterygium of pectoral girdle: (0) falling short of rostral node; (1) reaching rostral node.

39. Lateral prepelvic processes of pelvic girdle: (0) short to moderately long; (1) extremely long with acute tips; (2) extremely long with biramous tips.

40. Clasper glans: (0) little expanded; (1) expanded.

41. Clasper glans: (0) without component rhipidion; (1) with component rhipidion.

42. Clasper glans: (0) without component pela; (1) with component pela.

43. Clasper glans: (0) without component roll; (1) with component roll.

44. Clasper glans: (0) without component promontory; (1) with component promontory.

45. Clasper glans: (0) without component projection; (1) with component projection.

46. Ventral terminal cartilage: (1) with sharp, naked lateral margin; (2) with sharp naked terminal margin (3) without sharp naked margins.

47. Clasper glans: (1) with component eperon; (2) without component eperon.

48. Dorsal marginal cartilage: (0) with distal extension forming pseudorhipidion; (1) without distal extension and pseudosiphon absent.

49. Dorsal terminal 1 cartilage: (0) present; (1) absent.

50. Dorsal terminal 2 and 3 cartilages: (0) arranged in series; (1) arranged in parallel.

51. Clasper skeleton: (0) with three dorsal terminal cartilages; (1) with four dorsal terminal cartilages; (2) with two dorsal terminal cartilages.

52. Ventral terminal cartilage: (0) absent; (1) joined proximally with distal margin of ventral marginal or accessory terminal 1 cartilage; (2) spoon-shaped and without antero-medial or distal medial process; (3) with antero-medial notch and/or distal medial process.

53. Ventral terminal cartilage: (0) absent; (1) without proximal medial expansion; (2) with proximal medial expansion. **Remarks**: in the original character of McEachran & Dunn (1998) the three statements were coded as (?), (0) and (1), respectively. Since TNT does not properly read this coding and no grouping information are added, we changed the statements as (0), (1) and (2), accordingly.

54. Ventral terminal and accessory terminal 1 cartilages: (0) not connected by ligament; (1) connected by ligament or by accessory terminal 3 cartilage; (2) connected by accessory terminal 3 cartilage.

55. Accessory terminal 1 cartilage: (0) with slender tip; (1) with distal disc-shaped expansion.

56. Accessory terminal 2 cartilage: (0) with slender tip; (1) with expanded disc-shaped tip.

57. Electrocytes: (0) without cortical processes; (1) with cortical processes.

58. Electrocytes: (0) longer than broad; (1) modified cup-shaped; (2) narrow, modified cupshaped; (3) intermediate shaped; (4) disc shaped.

59. Principal cusp: (0) erect; (1) oblique. **Remarks:** according to Herman *et al*. (1994, 1995, 1996) the principal cusp is always erect in all teeth at all jaw positions or sometimes slightly inclined in outgroups and most of the skate genera. The main cusp is on the contrary oblique toward the commissure in *Atlantoraja*, *Irolita*, *Psammobatis*, *Rhinoraja*, *Rioraja*, *Sympterygia*, *Anacanthobatis* (*Schroederobatis*), *Breviraja*, *Fenestraraja*, *Gurgesiella*, and *Malacoraja* Herman *et* *al*. (1994, 1995, 1996). *Ostarriraja* gen. nov. show the basal condition in which the small cusp is erect ot slightly inclined, whereas the condition is unknown in North Pacific and Amphi-American Assemblages, *Brochiraja* and *Dentiraja*.

60. Sexual heterodonty: (0) absent; (1) present. **Remarks**: this character is demonstrated by the different tooth morphology of male and female adult skates, mainly in the high of the main cusp. In case of presence of sexual heterodonty, the main cusp is very long in males, whereas it is significantly short or replaced by a transverse ridge in adult females (Herman *et al.* 1994, 1995, 1996). Sexual heterodonty has been reported for *Rhinobatos*, *Atlantoraja*, *Irolita*, *Pavoraja*, *Psammobatis*, *Rhinoraja*, *Rioraja*, *Sympterygia*, *Anacanthobatis* (*Schroederobatis*), *Breviraja*, *Cruriraja*, *Fenestraraja*, *Gurgesiella*, and *Malacoraja*, Neoraja, *Rajella*, *Raja* and *Brochiraja* (Herman *et al*. 1994, 1995, 1996). All the other taxa show sexual homodonty. However, juvenile males that in adult stages should show an elongate cusp, actually present the same condition of adult females (Herman *et al*. 1994, 1995, 1996). For this reason, being *Ostarriraja* gen. nov represented by a single juvenile female, the presence of sexual heterodonty is unknown. The condition is unknown also in *Zapterix*, *Trygonorrhina*, North Pacific and Amphi-American Assemblages, and *Dentiraja.*

61. Cusp height (in case of homodonty): (0) low; (1) high. **Remarks**: according to Herman *et al.* (1994, 1995, 1996) there are two kinds of tooth morphology in case of sexual homodonty: the principal cusp can be either low or high. The former condition is present in *Arhynchobatis*, *Notoraja*, *Pseudoraja*, *Rhinoraja*, *Springeria*, *Dipturus*, *Okamejei*, and *Rostroraja.* On the contrary, in case of homodonty a high main cusp is present in *Bathyraja*, *Amblyraja*, *Cruriraja*, *Dactylobatus*, and *Leucoraja*.

62. Labial cutting edge: (0) absent; (1) present. Remarks: Herman *et al*. (1994, 1995, 1996) describe the presence of a labial (outer) cutting edge in the following genera: *Rioraja*, *Sympterygia*, *Amblyraja*, *Anacathobatis* (*Schroederobatis*), and *Gurgesiella*. Outgroups and all the others skates, including *Ostarriraja* gen. nov., lack the labial cutting edge.

63. Tooth vascularization: (0) holaulacorhizid; (1) secondary hemiaulacorhizid. **Remarks**: according to Herman *et al*. (1994, 1995, 1996) skates mostly show a particular kind of tooth vascularization which is the secondary hemiaulacorhizid type (state 1). However, only *Rhinoraja* and *Rostroraja* among modern skates still retain the basal condition of vascularization (holaulacorhizid; state 0) typical of outgroups and *Ostarriraja* gen. nov.

64. Osteodentine: (0) absent; (1) present. **Remarks**: The phylogeny of Aschliman *et al.* (2012a) recovered the presence of osteodentine supportive of the monophyly of skates. However, osteodontine in large teeth was only detected in the roots of *Rhinoraja* and *Rostroraja* (Herman *et al.* 1994, 1995, 1996) and its presence is therefore not supportive of the clade in our phylogeny. This condition in *Ostarriraja* gen. nov. is unknown.

65. Lingual uvula: (0) present; (1) absent. **Remarks**: Most of the skates retain a small uvula in their lingual face, although not well-developed like in outgroups (state 0). The derived condition in which the uvula is lost (state 1), has been reported for *Arhynchobatis*, *Atlantoraja*, *Irolita*, *Notoraja*, *Pavoraja*, *Psammobatis*, *Pseudoraja*, *Rioraja*, *Sympterygia*, *Anacanthobatis* (*Schroederobatis*), *Cruriraja*, *Fenestraja*, *Okamejei*, *Rostroraja*, *Raja*, and some species of *Leucoraja* (Herman *et al*. 1994, 1995, 1996). *Ostarriraja* gen. nov. shows the derived condition (1, absence of lingual uvula).

66. Labial apron: (0) absent; (1) present. **Remarks**: A labial apron is present only in the following genera according to Herman *et al*. (1994, 1995, 1996): *Dipturus*, *Leucoraja* and *Rostroraja*. *Ostarriraja* gen. nov. shows the basal condition in which the apron is absent.

67. Root stem: (0) low; (1) high. **Remarks**: A high root stem has been described for *Bathyraja*, *Irolita*, *Notoraja*, *Pavoraja*, *Rhinoraja*, *Amblyraja*, *Cruriraja*, *Dactylobatus*, *Fenestraja*, *Gurgesiella*, *Malacoraja*, *Leucoraja*, *Neoraja*, *Okamejei*, *Brochiraja*, and some species of *Rioraja* (Herman *et al.* 1994, 1995, 1996). *Ostarriraja* gen. nov. shows the derived condition with a high root stem.

68. Multilobed root (in adult): (0) absent; (1) present. **Remarks**: most of the skates show the basal condition among batoids in which the root is bilobed. However, some adult skate genera present a weak root multilobation of lateral and posterior teeth, e.g. *Anacanthobatis* (*Schroederobatis*), *Fenestraja*, *Gurgesiella*, *Rajella*, and *Rostroraja* (Herman *et al.* 1994, 1995, 1996). Multilobation of the root mostly occurs in juvenile specimens of some taxa (Herman *et al.* 1996). However it seems that *Ostarriraja* gen. nov., also in its late juvenile stage, presents two unequally developed massive root lobes.

69. Root coating: (0) absent; (1) present. **Remarks**: The collar (= root coating of Herman *et al.* 1996) has been recognized at the base of the root in two outgroups (*Zapterix* and *Trygonorrhina*), *Notoraja*, *Pavoraja*, *Dactylobatus*, *Gurgesiella*, *Malacoraja*, *Leucoraja*, *Rajella*, *Brochiraja* and *Rostroraja* (Herman *et al.* 1994, 1995, 1996). A well-developed root coating is also present in *Ostarriraja* gen. nov.

70. Cutting edges: (0) concave; (1) convex. **Remarks**: most of the skates show the basal condition of outgroups in which the cutting edges are concavely arched (state 0). However, *Arhynchobatis*, *Atlantoraja*, *Bathyraja*, *Irolita*, *Rhinoraja*, *Sympterygia*, *Anacanthobatis* (*Schroederobatis*), *Breviraja,* and *Malacoraja*, show convexly arched cutting edges (Herman *et al.* 1994, 1995, 1996). *Ostarriraja* gen. nov. shows the basal condition with concave cutting edges.

71. Suprascapulae fused to the median crest of the synarcual, forming the pectoral arch: (0) absent; (1) present. **Remarks:** The articulation of suprascapulae with the pectoral girdle has been used as diagnostic character to distinguish the different batoid clades. Aschliman *et al.* (2012a) pointed out that in guitarfishes, suprascapulae articulate with the neural arches of the vertebrae that are directly posterior to the synarcual. Skates are unique among batoids in that the suprascapulae are fused to the median crest of the synarcual, forming the pectoral arch (see also Garman 1913; Claeson 2008, 2011). The character is therefore coded (0) for *Zapterix*, *Pseudobatos* and *Trygonorrhina*, and (1) for all skates, including *Ostarriraja* gen. nov..

72. Compound radial: (0) band-like and slightly expanded distally, articulating with several radial segments in parallel fashion; (1) rod-like and articulated with single radial segments in serial fashion. **Remarks**: This character is taken from Aschliman *et al.* (2012a) who recognized that the first pelvic (compound) radial is thickened, variously shaped, and variably associated with distal radial segments in rays. In most of the batoids (including electric rays, guitarfishes, thornbacks, panrays and stingrays) the compound radial is bandlike and slightly expanded distally, articulating with several radial segments in parallel fashion (Aschliman *et al.* 2012a). In all skates, including *Ostarriraja* gen. nov., the compound radial is more rod-like and articulates with single radial segments in a serial fashion (see Holst & Bone 1993, fig. 1; Lucifora & Vassallo 2002, fig. 2; Aschliman *et al*. 2012a).

73. Pelvic girdle condyles: (0) close together; (1) separated. **Remarks**: Distinctly separated pelvic girdle condyles for the compound radial and basipterygium, with several radials directly articulating with the pelvic girdle between the two condyles are diagnostic for skates, including *Ostarriraja* gen. nov., whereas in most of the batoids pelvic girdle condyles are close together (Lucifora & Vassallo 2002; Aschliman *et al*. 2012a).

74. External margin of mesopterygium: (0) more or less straight, not fused to radials; (1) undulated, not fused to radials; (2) highly sinuous, fused with articulating radial elements. **Remarks**: Jeong & Nakabo (2009) described a more or less straight external margin of mesopterygium, not fused to radials (0) for skate outgroups. A margin undulated, but still not fused to radials (1) is present in *Hongeo*, *Gurgesiella* and *Sympterygia*. Finally, the external margin is highly sinuous and fused to radials near their base in *Anacanthobatis*, *Amblyraja*, *Atlantoraja*, *Bathyraja*, *Breviraja*, *Brochiraja*, *Dactylobatus*, *Dipturus*, *Irolita*, *Malacoraja*, *Okamejei*, *Psammobatis*, *Raja*, *Rajella*, *Rhinoraja* and North Pacific assemblage. *Ostarriraja* gen. nov. is coded as having the basal condition (0).
