# Supplementary material for: A new Miocene skate from the Central Paratethys (Upper Austria): the first unambiguous skeletal record for the Rajiformes (Chondrichthyes: Batomorphii)
Source: J Syst Palaeontol. 2018 Oct 30;17(11):937–60. doi: 10.1080/14772019.2018.1486336 (PMC6510527; doi:10.1080/14772019.2018.1486336)
Supplement: Supplemental_Material_Tables_S1_and_S2.docx [file TJSP_A_1486336_SM3274.docx]

Supplementary information to the article:

**A new Miocene skate from Central Paratethys (Upper Austria): The first unambiguous skeletal record for the Rajiformes (Chondrichthyes: Batomorphii)**

Giuseppe Marramà^a^*, Ortwin Schultz^b^ and Jürgen Kriwet^a^

^a^ University of Vienna, Department of Palaeontology, Althanstrasse 14, 1090, Vienna, Austria

^b^ Natural History Museum Vienna, Department of Geology and Paleontology, Burgring 7, 1010 Vienna, Austria

* Corresponding author. Email: giuseppe.marrama@univie.ac.at; phone: ++43-1-4277/53530

**Table S1.** List of synapomorphies for each node depicted in Fig. 8. See the explanation of characters and states in Appendix A.

| **Node** | **Clade** | **Synapomorphies** |
| --- | --- | --- |
| A | Rajiformes | 29(1), 65(1), 71(1), 72(1), 73(1) |
| B | - | 63(1), 69(0), 74(2) |
| C | Arhynchobatidae | 33(1), 45(1), 59(1) |
| D | Riorajinae | 11(1), 18(0), 32(1), 34(1) |
| E | Arhynchobatinae | 25(1), 27(4), 28(1), 38(1) |
| F | - | 50(1), 65(0), 67(1) |
| G | - | 49(1), 51(2) |
| H | - | 19(0), 39(1) |
| I | - | 42(1), 56(1) |
| J | - | 23(1), 59(0), 60(0) |
| K | - | 27(5), 28(0), 31(1), 58(2), 70(0) |
| L | - | 9(0), 67(1), 69(1) |
| M | - | 60(1) |
| N | Rajidae | 23(1), 34(2), 40(1), 41(1), 57(1) |
| O | - | 25(1), 35(1), 65(0) |
| P | Gurgesiellinae | 31(1) |
| Q | - | 26(2), 27(2), 38(1) |
| R | - | 17(1), 30(1), 31(2), 35(0), 49(1), 68(1) |
| S | Amblyrajinae | 12(1), 22(0), 26(1), 27(1), 51(1), 53(2) |
| T | - | 29(0), 48(1) |
| U | - | 60(0) |
| V | - | 35(0), 69(1) |
| W | Rajinae | 11(1), 20(0), 29(0) |
| X | - | 10(1) |
| Y | - | 9(2), 18(0), 23(0), 39(2) |
| Z | - | 21(0), 37(1), 54(1) |
| AA | - | 55(1) |

**Table S2.** Meristic counts and dental characters useful to discriminate *Ostarriraja* gen. nov. from selected living skate genera. Data from McEachran & Compagno (1982), Herman *et al.* (1994, 1995, 1996), Jacob & Mceachran (1994), Carvalho *et al.* (2005), Last & Lim (2010), Last *et al.* (2010, 2016), Soto & Costa (2010), Last & Alava (2013), Last & Séret (2016). The list does not include *Springeria* and *Schroederobatis*, *Cruriraja*, *Sympterygia and Psammobatis* for the very different pelvic girdle morphology, which excludes *a priori* the assignment of the new skate to these taxa.

| **Family** | **Genus** | **Total predorsal vertebrae** | **Total pectoral radials** | **Principal cusp** | **Labial cutting edge** | **Uvula** | **Apron** | **Root stem** | **Multilobation** | **Root coating** | **Cutting edges** |
| --- | --- | --- | --- | --- | --- | --- | --- | --- | --- | --- | --- |
|  | ***Ostarriraja*** | **65-70** | **86** | **erect** | **absent** | **absent** | **absent** | **high** | **absent** | **present** | **concave** |
| RAJIDAE | *Amblyraja* | 70 | 70 | erect | present | present | absent | high | absent | absent | concave |
|  | *Beringraja* | 68-96 | 65-78 | ? | ? | ? | ? | ? | ? | ? | ? |
|  | *Breviraja* | 88-108 | 56-75 | oblique | absent | present | absent | low | absent | absent | convex |
|  | *Dactylobatus* | ? | ? | erect | absent | present | absent | high | absent | present | concave |
|  | *Dentiraja* | 62-84 | 70-89 | ? | ? | ? | ? | ? | ? | ? | ? |
|  | *Dipturus* | 68-96 | 80-104 | erect | absent | ? | present | low | absent | absent | concave |
|  | *Hongeo* | 81-85 | 87-90 | ? | ? | ? | ? | ? | ? | ? | ? |
|  | *Leucoraja* | 80-118 | 61-91 | erect | absent | absent/present | present | high | absent | present | concave |
|  | *Malacoraja* | 84-103 | 68-75 | oblique | absent | present | absent | high | absent | present | convex |
|  | *Neoraja* | 65-78 | 60-69 | erect | absent | present | absent | high | absent | absent | concave |
|  | *Okamejei* | 63-82 | 71-96 | erect | absent | absent | absent | high | absent | absent | concave |
|  | *Raja* | 45-86 | 70-100 | erect | absent | absent | absent | low | absent | absent | concave |
|  | *Rajella* | 52-99 | 55-84 | erect | absent | present | absent | low | present | present | concave |
|  | *Rostroraja* | 59-65 | 65-105 | erect | absent | absent | present | low | present | present | concave |
| ARHYNCHOBATIDAE | *Arhynchobatis* | 118 | ? | erect | absent | absent | absent | low | absent | absent | convex |
|  | *Atlantoraja* | ? | ? | oblique | absent | absent | absent | low | absent | absent | convex |
|  | *Bathyraja* | 82-131 | 71-102 | erect | absent | present | absent | high | absent | absent | convex |
|  | *Brochiraja* | 81-115 | 58-77 | ? | absent | present | absent | high | absent | present | concave |
|  | *Irolita* | 97-112 | 87-105 | oblique | absent | absent | absent | high | absent | absent | convex |
|  | *Notoraja* | 94-134 | 60-84 | erect | absent | absent | absent | high | absent | present | concave |
|  | *Pavoraja* | 88-113 | 64-77 | erect | absent | absent | absent | high | absent | present | concave |
|  | *Pseudoraja* | 100-106 | 84-85 | erect | absent | absent | absent | low | absent | absent | concave |
|  | *Rhinoraja* | 109-127 | ? | oblique | absent | present | absent | high | absent | absent | convex |
|  | *Rioraja* | ? | ? | oblique | present | absent | absent | high/low | absent | absent | concave |
